# Cost-Effectiveness of Community-Based TB/HIV Screening and Linkage to Care in Rural South Africa

**DOI:** 10.1371/journal.pone.0165614

**Published:** 2016-12-01

**Authors:** Jennifer A. Gilbert, Sheela V. Shenoi, Anthony P. Moll, Gerald H. Friedland, A. David Paltiel, Alison P. Galvani

**Affiliations:** 1 Department of Epidemiology of Microbial Diseases, Yale School of Public Health, New Haven, Connecticut, United States of America; 2 Center for Infectious Disease Modeling and Analysis, Yale School of Public Health, New Haven, Connecticut, United States of America; 3 Department of Medicine, Section of Infectious Diseases, AIDS Program, Yale University School of Medicine, New Haven, Connecticut, United States of America; 4 Church of Scotland Hospital, Tugela Ferry, KwaZulu-Natal, South Africa; 5 Department of Health Policy and Management, Yale School of Public Health, New Haven, Connecticut, United States of America; Rush University, UNITED STATES

## Abstract

**S**outh Africa has one of the highest burdens of TB worldwide, driven by the country’s widespread prevalence of HIV, and further complicated by drug resistance. Active case finding within the community, particularly in rural areas where healthcare access is limited, can significantly improve diagnosis and treatment coverage in high-incidence settings. We evaluated the potential health and economic consequences of implementing community-based TB/HIV screening and linkage to care. Using a dynamic model of TB and HIV transmission over a time horizon of 10 years, we compared status quo TB/HIV control to community-based TB/HIV screening at frequencies of once every two years, one year, and six months. We also considered the impact of extending IPT from 36 months for TST positive and 12 months for TST negative or unknown patients (36/12) to lifetime use for all HIV-infected patients. We conducted a probabilistic sensitivity analysis to assess the effect of parameter uncertainty on the cost-effectiveness results. We identified four strategies that saved the most life years for a given outlay: status quo TB/HIV control with 36/12 months of IPT and TB/HIV screening strategies at frequencies of once every two years, one year, and six months with lifetime IPT. All of these strategies were very cost-effective at a threshold of $6,618 per life year saved (the per capita GDP of South Africa). Community-based TB/HIV screening with linkage to care is therefore very cost-effective in rural South Africa.

## Introduction

South Africa has the highest incidence of TB/HIV co-infection in the world [1]. Recent efforts to mitigate the TB/HIV co-epidemic have focused on integrating TB and HIV control within the healthcare setting, including screening identified TB patients for HIV and HIV patients for TB, providing isoniazid preventive therapy (IPT) to individuals enrolled in HIV care, and expanding antiretroviral therapy (ART) eligibility to individuals with CD4+ cell counts below 500 cells per milliliter [1, 2]. However, access to healthcare in much of South Africa is limited due to insufficient resources, particularly in rural areas. Active case finding for TB and HIV within the community, compared to the currently implemented passive case finding that requires individuals to seek care themselves, has the potential to identify additional cases [3–9], thereby improving clinical outcomes and reducing transmission.

Studies have shown that community-based HIV testing with point-of-care CD4+ cell count testing is very cost-effective for HIV control in settings with high incidence, and can effectively link eligible HIV-positive individuals to ART [6, 8]. However, the combination of TB/HIV screening and linkage to TB and HIV care has not been previously evaluated. Integration of both TB and HIV control is particularly important in settings with high rates of co-infection, because the incidence of each disease is dependent on the incidence of the other. Diagnostic tests for TB are more expensive than HIV tests, and TB/HIV screening requires more provider and patient time than HIV screening alone, increasing the cost per person screened [10]. Beyond diagnosis, linkage to care requires determining eligibility for treatment (via CD4+ cell count for HIV and drug resistance testing for TB) and ensuring that patients enroll in the appropriate treatment, including IPT for patients with HIV. ART is important for both TB and HIV control, because HIV is a driver of TB disease in South Africa and 80% of TB cases are co-infected with HIV [9]. Integrated TB/HIV control is therefore requisite for addressing the TB/HIV co-epidemic.

To determine the cost-effectiveness of community-based TB and HIV screening and linkage to care in rural South Africa, we developed a mathematical model that incorporates the dynamics of both TB and HIV infection and treatment, as well as economic resource utilization and health burden. We parameterized the model with clinical and epidemiological data from South Africa to predict the number of TB, HIV, MDR-TB, and XDR-TB cases averted, as well as the number of life years saved, as a result of the screening program over a 10-year time horizon. We considered TB/HIV screening frequencies of once every two years, one year, and six months. Analyzed from the perspective of the South African Department of Health, we found that community-based TB/HIV screening with linkage to care was very cost-effective.

## Materials and Methods

### Mathematical model

To examine the potential impact of community-based TB/HIV screening and linkage to care over a 10-year period, we extended our previous model of TB and HIV transmission in a rural area of South Africa [9] to include healthcare costs and years of life saved. We additionally updated the model parameters to reflect the most current data for TB and HIV incidence and treatment coverage [11, 12]. We modeled drug-sensitive TB, MDR-TB, and XDR-TB, including acquired and amplified drug resistance, self-cure, strain fitness, exogenous re-infection, and mortality [13], and parameterized our model using data from clinical and epidemiological studies (Table 1 and Table I in S1 Text).

**Table 1:** Diagnostic, treatment, and cost parameters.

Parameter	Additional Specifications	Base Case Value (Range)	Reference
**Status quo TB diagnosis and treatment**			
Status quo case detection rate (per year)		72.5% (60–90%)	[11]
Bacteriologic coverage		80% (80–100%)	[29]
Proportion of TB cases that are infectious	HIV-	65%	[13]
	HIV+	30%	[13]
	HIV+ on ART	55%	[13]
Sensitivity symptom questionnaire	HIV-	69%	[48]
	HIV+	79%	[49]
Specificity symptom questionnaire	HIV-	61%	[48]
	HIV+	49.6%	[49]
Negative predictive value symptom questionnaire (assuming 5% TB prevalence among HIV+)	HIV+	97.84%	[2, 49]
Sensitivity Xpert for TB detection	Infectious TB	98.3%	[65]
	Noninfectious TB	76.9%	[65]
Specificity Xpert for TB detection		99.2% (98.2–99.7)	[65]
Sensitivity Xpert for rifampicin resistance detection		94.4%	[65]
Specificity Xpert for rifampicin resistance detection		98.1 (96.6–99%)	[65]
Sensitivity culture	Infectious TB	100%	[32]
	Noninfectious TB	68%	[32]
Specificity culture		100%	[32]
Efficacy of first-line treatment			
DS TB	HIV-	77%	[2]
	HIV+	69%	[2]
	HIV+ on ART	75%	[2, 26–28]
MDR-TB	HIV-	47%	[13, 16, 66, 67]
	HIV+	30%	[13, 16, 66, 67]
	HIV+ on ART	42%	[13, 16, 66, 67]
Efficacy of second-line treatment			
MDR-TB	HIV-	67%	[13, 16, 66–68]
	HIV+	45%	[13, 16, 66–68]
	HIV+ on ART	60%	[13, 16, 66–68]
XDR-TB	HIV-	54%	[13, 66, 69]
	HIV+	36%	[13, 66, 69]
	HIV+ on ART	49%	[13, 66, 69]
Default between drug resistant TB diagnosis and treatment initiation (proportion)	MDR-TB outpatient	15% (0–24%)	[32, 50]
	MDR/XDR-TB inpatient	50% (29–73%)	[33]
Default from TB treatment			
First-line and decentralized second-line	DS and MDR-TB	7% (0–50%)	[50, 70]
Inpatient second-line	XDR-TB	28% (0–50%)	[33, 71, 72]
**Status quo TB diagnostic costs (per patient)**			
Baseline screening		$43.12 (32.22–73.11)	[10, 73]
MDR/XDR-TB suspect		$118.18 (92.55–222.50)	[10, 73]
**TB treatment and healthcare costs (monthly)**			
First-line TB treatment and healthcare		$271.29 (269.54–274.49)	[10, 73]
Second-line TB treatment and healthcare			
MDR-TB		$298.30 (164.74–298.30)	[10, 73]
XDR-TB		$1,073.13 (518.10–1,073.13)	[10, 73]
**Status quo HIV diagnosis and treatment**			
Status quo ART coverage		52% (20–80%)	[38]
Default from ART		9.1% (0–50%)	[35]
Status quo HIV diagnostic costs (per patient)		$28.80 (22.92–28.80)	[8, 74]
**HIV treatment and healthcare costs (monthly)**			
Not in healthcare		$54.40 (54.40–93.19)	[74–77]
In healthcare, not on ART		$102.56 (102.56–141.35)	[74–77]
In healthcare, on ART		$78.49 (78.49–161.65)	[74–77]
**IPT**			
Percentage of eligible patients initiating IPT following screening		31% (20–80%)	[6, 35, 36]
Default from IPT		9.1% (0–50%)	[37]
Efficacy		100% (22–100%)	[15, 47]
Adherence		87% (21–87%)	[47, 78]
IPT Costs (monthly)		$3.30 (3.21–3.30)	[79]
**TB/HIV screening**			
Acceptance of screening		70% (25–100%)	[6, 8, 80, 81]
Percentage of eligible patients initiating ART and IPT following diagnosis		31% (20–80%)	[6, 8, 80, 81]
Percentage of ART ineligible patients initiating IPT following screening		31% (20–80%)	[6, 8, 80, 81]
Percentage of patients initiating first-line and decentralized second-line TB treatment following diagnosis		85% (50–100%)	[32]
Percentage of patients initiating inpatient second-line TB treatment following diagnosis		50% (0–70%)	[33]
**TB/HIV screening costs (per patient)**			
TB positive and HIV+		$205.30 (97.22–267.13)	[6, 8, 10]
TB positive		$165.84 (82.02–227.57)	[6, 8, 10]
HIV+		$101.19 (24.21–163.02)	[6, 8, 10]
TB negative and HIV-		$45.57 (10–120)	[6, 8, 10]

In line with other models of TB infection [14], latent infections are stratified into rapid or slower progression. Individuals progress from latent infection to active infectious or non-infectious TB disease. Non-infectious individuals can become infectious over time [15, 16], and consistent with clinical studies, a small percentage of smear-negative patients are categorized as infectious [17]. The effectiveness of TB treatment is determined by drug efficacy, adherence, and default [18]. Individuals who receive effective treatment recover from active disease, while individuals who are ineffectively treated remain infectious and are at risk for acquired or amplified resistance [19]. A small proportion of patients with active TB or ineffective treatment may also self-cure and return to a latently infected state [20]. Patients who successfully complete treatment and recover may relapse to active disease [20]. Both recovered and latently infected individuals can be exogenously re-infected with drug-sensitive or resistant TB, contingent upon partial immunity and the time-dependent risk of infection [21].

We modeled the early and late stages of HIV disease, mortality, and prevalence-dependent behavior change, as well as ART initiation and default. HIV-negative individuals can become infected with HIV at a rate proportional to the prevalence of HIV in the population [11, 22]. Consistent with clinical recommendations in 2015, HIV-infected individuals with CD4+ cell count below 500 cells per milliliter are eligible to initiate ART [23], but only a proportion of actually begin ART [11, 22]. HIV-infected individuals who are co-infected with active TB may also start ART, regardless of CD4+ cell count [23].

We take into account both the effect of HIV and ART on TB disease, as well as TB disease on HIV pathogenesis, such that disease progression and mortality rates depend on whether an individual is co-infected [24]. Overall, HIV infection increases the likelihood of progression to primary active disease in those infected with TB, the rate at latent infection reactivates to active disease, and mortality from TB disease [1]. Infection with HIV also reduces the efficacy of TB treatment, as well as partial immunity to superinfection or reinfection with another TB strain [1]. Similarly, active TB disease decreases CD4+ cell counts, speeds up progression to AIDS, and increases mortality [24, 25]. These exacerbations between TB and HIV are mitigated by ART [26–28].

### Intervention strategies

We compared the impact of community-based TB/HIV screening and linkage to care over a 10-year time horizon on the disease incidence, mortality, and healthcare costs arising from drug-sensitive TB, MDR-TB, XDR-TB and HIV. Specifically, we compared a total of eight mutually exclusive strategies (Table 2).

**Table 2:** Intervention strategies evaluated.

Strategy	TB/HIV Control	IPT Duration
Status Quo, IPT 36/12	Status quo	36/12 months
Status Quo, IPT life	Status quo	Lifetime
Screen 2 yr, IPT 36/12	Community-based TB/HIV screening every 2 years	36/12 months
Screen 2 yr, IPT life	Community-based TB/HIV screening every 2 years	Lifetime
Screen 1 yr, IPT 36/12	Community-based TB/HIV screening every year	36/12 months
Screen 1 yr, IPT life	Community-based TB/HIV screening every year	Lifetime
Screen 6 mo, IPT 36/12	Community-based TB/HIV screening every 6 months	36/12 months
Screen 6 mo, IPT life	Community-based TB/HIV screening every 6 months	Lifetime

#### Status quo TB and HIV detection and treatment

We evaluated the effectiveness of community-based TB/HIV screening and linkage to care relative to status quo TB and HIV control currently implemented in rural South Africa. In the baseline scenario, individuals with TB and/or HIV symptoms self-present to a healthcare facility for symptom detection (*e*.*g*. cough lasting longer than two weeks, fever, night sweats, and/or weight loss), Xpert MTB/RIF (a newer, fully automated, cartridge-based technology to rapidly diagnose both TB disease and rifampicin resistance), sputum smear, and/or chest x-ray. We assume the status quo TB case detection rate to be 72.5% [11]. The bacteriologic coverage (*i*.*e*. the percentage of suspected TB patients who receive microbiologic testing) for KwaZulu-Natal is estimated to be 80% [29]. Per South African treatment guidelines [3], patients identified as having rifampicin-sensitive active TB disease through Xpert, as well as patients who do not receive microbiologic testing but are suspected of having active TB disease, are enrolled in first-line therapy (the administration of four oral antibiotics for two months and two antibiotics for at least an additional four months). Any TB patients remaining symptomatic and/or smear positive after two to three months of first-line therapy are suspected of TB drug resistance, and then assessed by culture and drug susceptibility testing (DST) [30]. Although sputum culture is the gold standard for diagnosis of active pulmonary TB disease, it can require over three months to receive the results of culture combined with DST [31, 32]. Patients who have MDR or XDR-TB confirmed are then started on second-line therapy for at least 24 months. Patients who are initially identified through Xpert as having rifampicin-resistant active TB disease are also started on second-line therapy while awaiting confirmation by DST. Current second-line TB treatment guidelines recommend home-based treatment for MDR-TB management whenever possible, where patients are visited by a nurse for the administration of injectable drugs and supervision of oral medications over a 24-month period [33]. XDR-TB patients are hospitalized for at least the intensive phase of their treatment [30, 33].

Following HIV diagnosis, individuals are referred to an HIV clinic to receive a CD4+ cell count to determine their eligibility for ART, but it is estimated that 15% to 45% of patients do not follow-up for this evaluation [34, 35]. Of those who are determined to be ART-eligible (CD4+ cell count ≤ 500 cells per milliliter), only around half start treatment [35, 36], and about 25% of those individuals default within three years [37]. We assume the current coverage of ART and IPT to be 52% [38–40]. While the current South African guidelines recommend ART for individuals with a CD4+ cell count below 500 cells per milliliter, we additionally considered the scenario where all HIV-infected individuals were eligible for ART regardless of CD4+ cell count, as has recently been demonstrated to be effective at reducing overall HIV incidence in a population [39, 40].

The South African Department of Health recommends 36 months of IPT for TST positive individuals and 12 months for TST negative or unknown individuals (from hereon forth abbreviated as 36/12 months) [34, 41, 42] to treat latent infection and prevent progression to active TB [43]. IPT is typically initiated simultaneously with ART [44, 45]. We additionally considered the scenario where IPT was given to HIV-positive individuals for the entire duration of their lives, as has been recently recommended by the WHO [46]. We assumed individuals treated with IPT and adherent to treatment were protected from infection with or latent reactivation of drug-susceptible TB for the duration of the IPT, dependent on treatment efficacy (22–100%) and patient adherence (21–87%), and that IPT does not prevent the reactivation or transmission of drug resistant TB [15, 42, 47]. For the status quo scenario, we additionally assumed that only patients who initiated ART received IPT, as is currently implemented in South Africa [45], and that all patients on ART receive IPT. Upon IPT completion, individuals return to baseline relative risk of slower TB reactivation and infection [41].

#### Community-based TB/HIV screening and linkage to care

We model a community-based TB/HIV screening and linkage to care program that combines HIV testing and counseling with TB testing [9]. TB/HIV screening is offered and administered to any individual within the community who accept TB or HIV testing, with acceptance rates between 70% and 100% [6, 8]. As the first step in this community-based intervention, a questionnaire is used to screen individuals for TB symptoms (sensitivity of 69–79%) [48, 49]. Sputum is then collected from symptomatic individuals for Xpert, culture, and DST (sensitivity of 68–100%) [32]. Individuals diagnosed by Xpert and/or culture are linked to the appropriate first- or second-line TB treatment, with 85% of individuals starting treatment [32, 50]. Currently Xpert technology can only detect rifampicin resistance, and thus XDR-TB diagnoses can only be made after waiting two months for the DST results. We additionally considered the scenario where Xpert could detect resistance to second-line drugs and therefore be used to rapidly diagnose XDR-TB. Individuals are also given a rapid HIV antibody test (sensitivity of 98.2–100%) [51]. Those who test positive have their result confirmed by a second rapid HIV test, and then have a point-of-care CD4+ cell count performed to immediately determine their ART eligibility status. If eligible, patients are linked to their local ART clinic for ARV treatment and IPT initiation, with approximately 31% of individuals identified as eligible initiating ART and IPT [6, 8]. We assume individuals with a CD4+ cell count less than 500 cells per milliliter to be eligible for ART, as has been recommended by South African ART guidelines [52]. We additionally considered the scenario where all HIV-infected individuals were eligible for ART, irrespective of CD4+ cell count [39, 40].

Individuals not yet eligible for ART (with a CD4+ cell count greater than 500 cells per milliliter) are linked to their local ART clinic for IPT, as well as regular CD4+ cell count monitoring [6, 8]. We considered two different approaches to IPT administration: a) 36/12 months of IPT, reflecting current South African guidelines for IPT administration; and b) lifetime IPT for all HIV patients, reflecting recent changes to WHO recommendations and findings that lifetime IPT may greatly reduce TB burden [46, 53].

The WHO states that there is not sufficient evidence to conclude that IPT administration increases MDR-TB incidence via the generation of isoniazid mono-resistance [46], so we assume that neither status quo IPT nor an increase in IPT coverage via the TB/HIV screening intervention will increase the incidence of MDR-TB. However, some predictions have been made that isoniazid mono-resistance may become highly prevalent after 50 or more years of IPT, thus increasing the burden of MDR and/or XDR-TB [15, 54]. Although the time horizon for our analysis is only 10 years, in a sensitivity analysis we also consider the “worst case” scenario whereby the community-based TB/HIV screening immediately causes 100% of the modeled population to become resistant to isoniazid, thus decreasing the efficacy of first-line TB treatment from 77% to 65% and increasing the probability that first-line treatment failures acquire MDR-TB from 3.8% to 61% [55]. While this worst case scenario is unrealistically pessimistic, and thus is not proposed as a likely possibility, it provides an upper bound on any potential increases in MDR- and XDR-TB incidence and thus on the costs that could arise from expanding IPT coverage.

Each modeled TB/HIV screening team consists of a professional nurse, two field health workers, and three counselors who screen between 1,800 and 4,800 individuals annually at community sites [9], costing between $61.83 and $23.18 per person screened, including the cost of a TB symptom questionnaire and rapid HIV test. We conservatively assume $61.83 per person screened as our base case cost, but vary the screening cost in the sensitivity analysis. In some settings, trained community health workers have been comparably effective at testing and linking individuals to HIV care as counselors, totaling as little as $6 per person screened [6]. In the sensitivity analysis, we additionally vary the rates of linkage to care to reflect any differences that might arise in other settings. The South African Department of Health aims to screen all individuals for TB once per year [3], which we incorporate as the base case, but also consider screening frequencies of once every two years, once every year, and once every six months.

### Health outcomes and costs

We considered the lifetime costs of TB and HIV detection and treatment among 90,000 adults in a rural South African setting. We performed the analysis over a 10-year time horizon from the perspective of the South African Department of Health, which is typically responsible for all medical costs in rural settings. Health burden was estimated with regard to the number of life years saved by an intervention strategy over the course of the lifetimes of the individuals in the population modeled. Costs and life years were discounted at an annual rate of 3%, following WHO guidelines [56]. Costs were presented in 2015 US dollars (US$).

### Cost-effectiveness

We calculated the incremental cost-effectiveness ratio (ICER) of the community-based TB/HIV screening and linkage to treatment strategies detailed above. The ICER of each strategy measures the additional cost per life year saved as the frequency of TB/HIV screening and/or the duration of IPT is increased. In accordance with WHO guidelines, we classified an intervention strategy as “very cost-effective” if its incremental cost-effectiveness ratio (ICER) was less than the South African per capita GDP in 2015 ($6,618) and as “cost-effective” if the ICER was less than three times the per capita GDP ($19,854) [56, 57]. Strategies with ICERs below the threshold for cost-effectiveness can be considered to be preferred or “economically efficient” strategies. Given that multiple strategies may be classified as cost-effective, the choice regarding which strategy should be implemented will ultimately depend on the Department of Health’s “willingness-to-pay” for each additional year of life saved. A strategy is considered “dominated,” (*i*.*e*. not optimal) if it costs more than an alternative strategy that is as or more effective.

### Uncertainty and sensitivity analysis

To assess the impact of parameter uncertainty on our cost-effectiveness analysis and to estimate the likelihood that a strategy would be optimal at a given willingness-to-pay threshold, we performed a probabilistic sensitivity analysis [58]. We assigned probability distributions to all parameters and costs by fitting the 95% confidence interval of beta or gamma distributions to plausible ranges from clinical and epidemiological data (ranges are shown in Table 1 and Table I in S1 Text; uncertainty distributions are specified in Table II in S1 Text). Samples were drawn from these parameter distributions 1,000 times using Latin hypercube sampling and run through the model to project distributions of intervention cost and life years saved for each strategy. Net health benefits were calculated from the cost and life years saved distributions as the difference between the average health benefit of an intervention strategy (*i*.*e*. life years saved) and the average intervention and healthcare costs, divided by the threshold cost-effectiveness ratio [59]. We calculated the net health benefit of each strategy across a range of willingness-to-pay thresholds. From this, we found the probability that a given scenario had the greatest net health benefit compared to its alternatives at a given level of willingness-to-pay. We used these probabilities to generate cost-effectiveness acceptability curves that quantified the uncertainty surrounding our cost-effectiveness ratio estimates. The acceptability curves demonstrate the likelihood that a given intervention strategy saved the largest number of life years for a given outlay (*i*.*e*. was optimal at a given willingness-to-pay threshold).

Considering South Africa’s goal of screening all individuals once per year, we also performed a one-way sensitivity analysis to determine the impact of parameters on the cost-effectiveness ratio of annual TB/HIV screening. Specifically, we calculated the ICER of annual TB/HIV screening with 36/12 months of IPT and lifetime IPT at the minimum and maximum value of each parameter (Table 1 and Table I in S1 Text) to determine whether the strategy remained cost-effective at these extreme values.

## Results

### Epidemiological impacts

**Fig 1:**
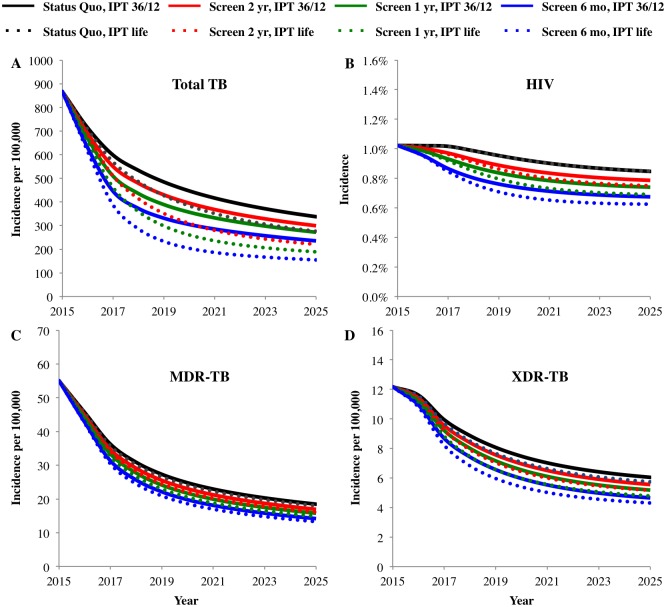
Epidemiological impact. Epidemiological impact of community-based TB/HIV screening and linkage to care at frequencies of once every two years (Screen 2 yr), once every year (Screen 1 yr), and once every six months (Screen 6 mo) relative to status quo with 36/12 months of IPT (IPT 36/12) or lifelong IPT (life IPT) on (A) total TB incidence per 100,000 population, (B) HIV incidence (%), (C) MDR-TB incidence, and (D) XDR-TB incidence over 10 years. Data points can also be found in Table III in S1 Text.

Under status quo TB/HIV control—including Xpert implementation, 36/12 months of IPT for HIV-infected individuals on ART, and MDR-TB care decentralization—annual total TB incidence would be reduced from 868 per 100,000 to 298 cases per 100,000 population (Fig 1A and Table III in S1 Text) after 10 years. With the extension of ART eligibility in 2015 to individuals with a CD4+ cell count below 500 cells per milliliter, annual HIV incidence was reduced over 10 years from 1% to 0.8% (Fig 1B and Table III in S1 Text). Annual MDR-TB incidence was similarly reduced, from 54 to 16 cases per 100,000 population (Fig 1C and Table III in S1 Text), and annual XDR-TB incidence from 12 to 5 cases per 100,000 population (Fig 1D and Table III in S1 Text). Expanding IPT duration to lifetime further reduced annual total TB incidence to 254 cases per 100,000 population after 10 years (Fig 1A and Table III in S1 Text), but had a negligible impact on MDR-TB, XDR-TB, and HIV incidence (Fig 1B–1D and Table III in S1 Text).

When the community-based TB/HIV screening and linkage to care intervention was implemented under current 36/12 months IPT guidelines, annual total TB incidence was reduced to between 274 per 100,000 to 233 cases per 100,000 population, for screening frequencies between once every two years and every six months (Fig 1A and Table III in S1 Text). Annual HIV incidence was reduced to between 0.8% and 0.6% (Fig 1B and Table III in S1 Text), corresponding to screening frequencies between once every two years to once every six months. Annual MDR-TB incidence was reduced to between 15 and 14 MDR-TB cases per 100,000 population (Fig 1C and Table III in S1 Text), while annual XDR-TB incidence fell to between 5 and 4 XDR-TB cases per 100,000 population (Fig 1D and Table III in S1 Text). When IPT was extended to the lifetime of HIV patients, TB/HIV screening and linkage to care further reduced total TB incidence to between 208 and 153 cases per 100,000 population after 10 years (Fig 1A and Table III in S1 Text), while negligibly impacting HIV, MDR-TB, and XDR-TB relative to TB/HIV screening with 36/12 months of IPT (Fig 1B–1D and Table III in S1 Text).

### Cost-effectiveness analysis

We first estimated the additional discounted costs associated with community-based TB/HIV screening and linkage to care, as well as the discounted life years (LYs) saved, at screening frequencies of once every two years, one year, and six months, relative to status quo (Table 3). Under status quo TB/HIV control with 36/12 months of IPT, health costs were projected to be $225 million with 1.86 million LYs saved over the next 10 years. Extending IPT duration to lifetime cost an additional $7.7 million and added approximately 3,000 LYs. Implementing community-based TB/HIV screening and linkage to care, when 36/12 months of IPT were recommended, cost an additional $25.8 million to $97.3 million for screening frequencies between once every two years and once every six months, while also gaining between 11,600 and 34,500 LYs, respectively. Adding TB/HIV screening to lifetime IPT cost between $32.6 million and $108.3 million more than status quo with lifetime IPT, while gaining another 20,600 to 51,400 LYs for screening frequencies of once every two years and once every six months, respectively.

**Table 3:** Cost-effectiveness analysis. Ten year cumulative TB and HIV infections, discounted lifetime costs, discounted lifetime benefits (life years saved), and ICERs for community-based screening and linkage to care with IPT duration of 36/12 months (IPT 36/12) and lifelong IPT (IPT life) for screening frequencies once every two years (Screen 2 yr), every one year (Screen 1 yr), and six months (Screen 6 mo). Modeled population size is approximately 90,000 (a typical rural community in South Africa). LY = life years.

	Total TB Cases	DS TB Cases	MDR TB Cases	XDR TB Cases	HIV Cases	Discounted Lifetime Costs (2015 US$)	Discounted Lifetime LYs	ICER ($/LY)
**All Strategies**								
**Status Quo, IPT 36/12**	4,189	3,878	241	71	8,359	225,249,000	1,861,000	-
**Status Quo, IPT life**	3,718	3,414	236	68	8,368	232,934,000	1,864,000	Weakly Dominated^a^
**Screen 2 yr, IPT 36/12**	3,795	3,501	227	67	7,863	251,067,000	1,873,000	Weakly Dominated^a^
**Screen 2 yr, IPT life**	3,167	2,883	220	63	7,641	265,509,000	1,885,000	1,700
**Screen 1 yr, IPT 36/12**	3,494	3,214	217	64	7,471	275,685,000	1,882,000	Strongly Dominated^b^
**Screen 1 yr, IPT life**	2,797	2,529	209	60	7,143	293,197,000	1,898,000	2,000
**Screen 6 mo, IPT 36/12**	3,067	2,807	201	59	6,894	322,515,000	1,895,000	Strongly Dominated^b^
**Screen 6 mo, IPT life**	2,336	2,088	193	55	6,513	341,250,000	1,915,000	2,800
**IPT 36/12 Months Strategies Only**								
**Status Quo, IPT 36/12**	4,189	3,878	241	71	8,359	225,249,000	1,861,000	**-**
**Screen 2 yr, IPT, 36/12**	3,795	3,501	227	67	7,863	251,067,000	1,873,000	2,200
**Screen 1 yr, IPT 36/12**	3,494	3,214	217	64	7,471	275,685,000	1,882,000	2,700
**Screen 6 mo, IPT 36/12**	3,067	2,807	201	59	6,894	322,515,000	1,895,000	3,400
**Lifelong IPT Strategies Only**								
**Status Quo, IPT life**	3,718	3,414	236	68	8,368	232,934,000	1,864,000	-
**Screen 2 yr, IPT life**	3,167	2,883	220	63	7,641	265,509,000	1,885,000	1,600
**Screen 1 yr, IPT life**	2,797	2,529	209	60	7,143	293,197,000	1,898,000	2,000
**Screen 6 mo, IPT life**	2,336	2,088	193	55	6,513	341,250,000	1,915,000	2,800

^a^By convention, a strategy is considered “Weakly Dominated” if it costs more and is less effective than some combination of other strategies. Both the “Status Quo, IPT life” and “Screen 2 yr, IPT 36/12” strategies are weakly dominated by the combination of the “Status Quo, IPT 36/12” and “Screen 2 yr, IPT life” strategies.

^b^By convention, a strategy is considered “Strongly Dominated” if it costs more and is less effective than some other strategy. The “Screen 1 yr, IPT 36/12” strategy is strongly dominated by the “Screen 2 yr, IPT life” strategy. “The “Screen 6 month, IPT 36/12” strategy is strongly dominated by the “Screen 1 yr, IPT life” strategy.

Cost-effectiveness analysis (Table 3) identified four “economically efficient” strategies (*i*.*e*. strategies which conferred the maximal benefit for a given outlay): status quo with 36/12 months of IPT; TB/HIV screening every two years with lifetime IPT; TB/HIV screening every year with lifetime IPT; and TB/HIV screening every six months with lifetime IPT. All three TB/HIV screening strategies involving 36/12 months of IPT were dominated relative to the TB/HIV screening strategies with lifetime IPT (*i*.*e*. they cost more and conferred fewer life years than the combination of lifetime IPT strategies), as was status quo detection with lifetime IPT relative to status quo detection with 36/12 months of IPT. ICERs estimated for the four efficient strategies were all significantly below the South African per capita GDP of $6,618, suggesting that even the most frequent program of TB/HIV screening with lifetime IPT would be very cost-effective by South African standards. When considered separately from strategies with lifetime IPT, all three screening strategies involving 36/12 months of IPT were very cost-effective at a threshold of $6,618.

**Fig 2:**
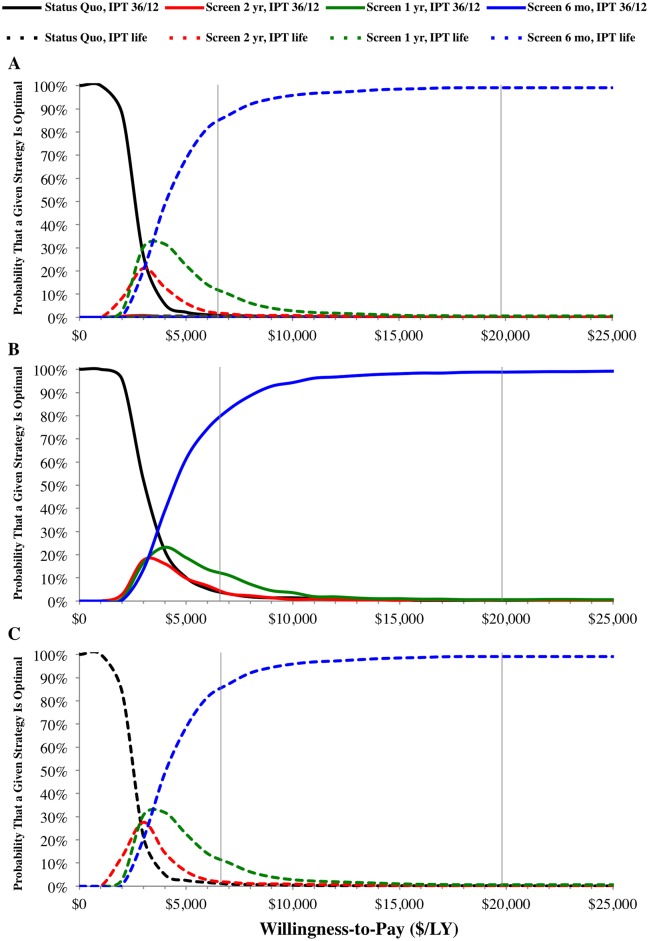
Probabilistic sensitivity analysis. Acceptability curves show the probability that a given strategy provides the greatest net health benefit at a given willingness-to-pay threshold (*i*.*e*. probability that the strategy is optimal) for (A) all strategies, (B) only strategies with 36/12 months of IPT, and (C) only strategies with lifetime IPT. Solid grey vertical lines indicate the thresholds $6,618 and $19,854 for “very cost-effective” and “cost-effective”, respectively. The solid red, green, and blue lines in panel (A) represent very low percentage values, and thus are close to zero.

In the probabilistic sensitivity analysis, we calculated the probability that a given strategy was optimal (*i*.*e*. maximizing net health benefits and number of life years saved for a given outlay) relative to the other strategies at different willingness-to-pay thresholds (Fig 2). Considering strategies with 36/12 months of IPT or lifetime IPT together (Fig 2A), status quo TB/HIV control with 36/12 months of IPT had the highest probability of being the most cost-effective option for a willingness-to-pay below $3,000 per LY. For a willingness-to-pay between $3,000 and $3,5000 per LY, annual TB/HIV screening and linkage to care with lifetime IPT had the highest probability of being the most cost-effective strategy. TB/HIV screening and linkage to care every six months with lifetime IPT had the highest probability of being the most cost-effective strategy for a willingness-to-pay above $3,500 (Fig 2A). Considering strategies with 36/12 months of IPT alone (Fig 2B), status quo had the highest probability of being the most cost-effective option for a willingness-to-pay below $4,000 per LY. For a willingness-to-pay above $4,000 per LY, TB/HIV screening at a frequency of six months had the highest probability of being the most cost-effective strategy. Considering just strategies with lifetime IPT (Fig 2C), status quo TB/HIV control had the highest probability of being the most cost-effective option at a willingness-to-pay below $3,000 per LY. For a willingness-to-pay between $3,000 and $3,500 per LY, annual TB/HIV screening with 36/12 months of IPT was optimal, while TB/HIV screening every six months with 36/12 months of IPT was optimal at a willingness-to-pay above $3,500 per LY.

**Fig 3:**
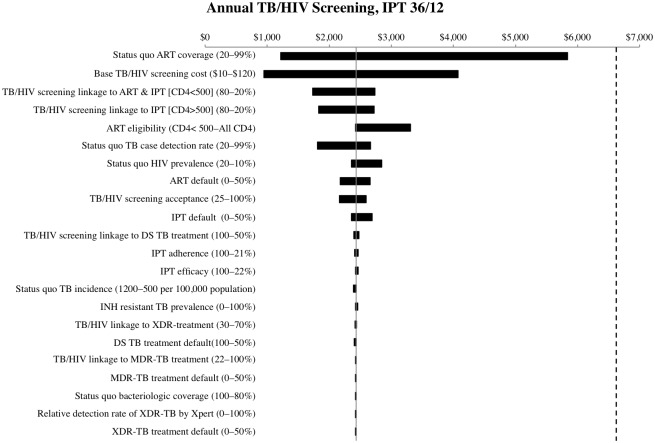
One-way sensitivity analysis: Standard IPT duration 36/12 months. One-way sensitivity analysis of cost-effectiveness ratio of annual TB/HIV screening and linkage to care relative to status quo, with standard IPT duration 36/12 months. The dashed vertical line indicates the South African per capita GDP threshold of $6,618 for “very cost-effective”. Ranges across which parameters were varied are indicated in parentheses.

**Fig 4:**
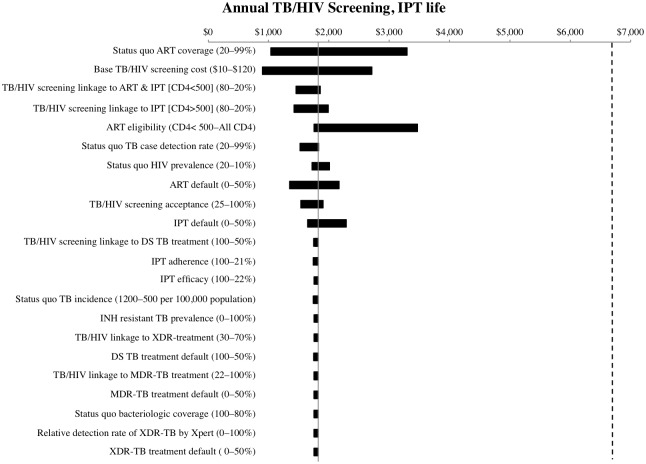
One-way sensitivity analysis: Lifelong IPT duration. One-way sensitivity analysis of cost-effectiveness ratio of annual TB/HIV screening and linkage to care relative to status quo, with lifelong IPT duration (IPT life). The dashed vertical line indicates the South African per capita GDP threshold of $6,618 for “very cost-effective”. Ranges across which parameters were varied are indicated in parentheses.

Using one-way sensitivity analysis, we investigated the extent to which the ICER for annual TB/HIV screening and linkage to care was impacted by variation in parameters (Figs 3 and 4). For both 36/12 months and lifetime IPT scenarios, the cost-effectiveness ratio of the screening intervention was most sensitive to status quo ART coverage. The cost-effectiveness of screening was also sensitive to the cost per person screened (excluding the cost of TB and HIV tests), status quo HIV prevalence, and the proportion of patients linked to HIV treatment and care. For all parameters varied, the intervention remained very cost-effective at a threshold below $6,618 (*i*.*e*. the per capita GDP). The ICER of annual TB/HIV screening was not sensitive to variation in TB or IPT parameters, including status quo TB incidence or linkage to TB care. We found that even if expanded IPT coverage increased isoniazid resistance or the sensitivity of Xpert for detecting XDR-TB was improved, community screening interventions would remain cost-effective. Finally, TB/HIV screening remained very cost-effective when ART eligibility was expanded to all HIV-infected individuals.

## Discussion

We found that community-based TB/HIV screening with linkage to care is a very cost-effective approach for reducing the burden of both diseases in South Africa. When the intervention was implemented over 10 years, total TB incidence was reduced to as low as 298 cases per 100,000 population and HIV incidence to 0.8%, while simultaneously decreasing both MDR and XDR-TB incidence. For the country as a whole, this integrated community-based intervention could save as many as 31.8 million life years.

The intervention remained very cost-effective even when status quo guidelines extended IPT from 36/12 months to lifetime or expanded ART eligibility from CD4+ cell count below 500 cells per milliliter to all HIV-infected individuals. Although we found that screening strategies of once every two years, once every year, or once every six months were all cost-effective, annual screening is most practical for implementation in rural settings. Accordingly, an aim of the South African Department of Health is to screen individuals for TB on an annual basis.

Although the intervention remained very cost-effective across entire ranges of plausible values, its cost-effectiveness was most influenced by variation in cost per person screened, as well as status quo HIV prevalence and ART coverage. In such settings where ART coverage is high or HIV prevalence is low, a smaller pool of untreated individuals is available in the community. Therefore a lower return for screening effort would be expected, thus increasing the cost per HIV positive or TB positive diagnosed and linked to care. In general, community-based TB/HIV screening is best suited to settings with high prevalence of both diseases, and where treatment coverage is suboptimal, as is the case currently in South Africa and many parts of sub-Saharan Africa.

Although increasing the cost per person screened unsurprisingly reduced the efficiency of the intervention, the ICER remained below the cost-effectiveness threshold for cost-effectiveness even if screening costs were doubled beyond our base costs parameterized from pilot implementation in rural South Africa. It is not until screening costs exceed 10 times the base cost that the intervention would no longer be cost-effective. We found the cost-effectiveness of annual TB/HIV screening to be robust to variation in TB incidence or treatment parameters, as well as IPT efficacy and adherence parameters. Because TB treatment costs constitute a fraction of total TB/HIV related healthcare costs, even when accounting for expensive drug resistant TB diagnosis and treatment costs, the costs of integrated TB/HIV healthcare will not be substantially affected by changes in TB costs alone. Furthermore, the cost-effectiveness of the community-based TB/HIV screening and linkage to care program was also unaffected by changes in status quo XDR-TB detection. While a smaller pool of undetected XDR-TB cases would be expected if improved Xpert technology were available for status quo XDR-TB diagnosis, overall status quo health care costs would also rise as additional XDR-TB cases are treated and the relative increase in cost per XDR-TB case diagnosed by the intervention would not push the ICER past the South African cost-effectiveness threshold.

Overall, screening strategies with lifetime IPT dominated strategies of the same frequency shorter IPT duration, supporting a South African policy change to extend IPT to lifetime. The benefit of lifetime IPT with status quo TB/HIV control can be attributed to the reduction in drug-sensitive TB incidence achieved by extending IPT duration. Furthermore, lifetime IPT also improves the impact of the community-based TB/HIV screening intervention in two ways. Firstly, TB incidence is reduced via expanded IPT coverage in a population with higher CD4+ cell counts who would otherwise not receive IPT. Secondly, patients with high CD4+ cell counts are identified and engaged in HIV care at an earlier stage of disease, minimizing the delay in initiating ART after becoming eligible. This reduces HIV incidence, because patients successfully treated with ART are much less likely to transmit HIV. Although this benefit was slightly diminished when status quo ART eligibility was extended to all HIV-infected individuals, community-based TB/HIV screening remained very cost-effective.

As with other modeling studies, the validity of our analysis is limited by the data available from epidemiological and clinical studies for parameterization. Nonetheless, sensitivity and uncertainty analyses where all model parameters were varied across plausible ranges demonstrated that our cost-effectiveness results are robust to parameter uncertainty. TB and HIV control in South Africa has also been rapidly evolving over the past decade, so “status quo” control strategies, guidelines, and technologies are likely to continue improving. Therefore, we evaluated the two most likely scenarios for South African policy change: increasing the duration of IPT administration to lifetime and expanding ART eligibility to all HIV-infected individuals, as have been recently recommended by the WHO. We additionally explored the impact of high status quo TB and HIV treatment coverage on our cost-effectiveness results in the sensitivity analysis.

Our results support community-based TB/HIV screening with linkage to TB and HIV care. This intervention has the potential to substantially reduce TB and HIV incidence simultaneously by expanding ART, IPT, and both first- and second-line TB treatment coverage. A number of recent studies have shown that home or community-based HIV testing and counseling is very effective and cost-effective in rural South Africa, with the potential to substantially reduce HIV incidence and associated mortality [6, 60–64]. These studies have established that a decentralized approach to HIV testing is not only both feasible and well accepted by patients, but that linkage to care and ART initiation occurs with subsequent virologic suppression. Our study also supports community-based HIV testing and counseling, while further demonstrating that combining TB screening with community-based HIV screening and linkage to care comprehensively addresses the TB/HIV co-epidemics of rural South Africa. In addition, community-based TB/HIV screening with linkage to care remains cost-effective under many different scenarios, including policy changes that would increase IPT duration to the lifetime of patients or make all individuals eligible for ART. The efficiency of this integrated community intervention would therefore be robust to changes in the South African TB/HIV healthcare landscape, and would likely have applicability beyond South Africa to other rural settings with high TB and HIV burdens.

## Supporting Information

S1 Text Technical Appendix. Supplementary methods, figures, and tables.
(DOCX)
