# Supplementary material for: Cost-Effectiveness of Community-Based TB/HIV Screening and Linkage to Care in Rural South Africa
Source: PLoS One. 2016 Dec 1;11(12):e0165614. doi: 10.1371/journal.pone.0165614 (PMC5131994; doi:10.1371/journal.pone.0165614)
Supplement: S1 Text — Supplementary methods, figures, and tables. (DOCX) [file pone.0165614.s001.docx]

**Cost-Effectiveness of Community-Based TB/HIV Screening and Linkage to Care in Rural South Africa**

**SUPPLEMENTARY APPENDIX**

**Jennifer A. Gilbert^1,2*^, Sheela V. Shenoi^3^, Anthony P. Moll^3,4^, Gerald H. Friedland^1,3^,**

**A. David Paltiel^5^, and Alison P. Galvani^1,2^**

**^1^** Department of Epidemiology of Microbial Diseases, Yale School of Public Health, New Haven, CT, USA

**^2^** Center for Infectious Disease Modeling and Analysis, Yale School of Public Health, New Haven, CT, USA

**^3^** Department of Medicine, Section of Infectious Diseases, AIDS Program, Yale University School of Medicine, New Haven, CT, USA

**^4^** Church of Scotland Hospital, Tugela Ferry, KwaZulu-Natal, South Africa

**^5^** Department of Health Policy and Management, Yale School of Public Health, New Haven, CT, USA

E-mail: jennifer.gilbert@aya.yale.edu (JAG)

**Additional Methods**

**Mathematical model**

To examine the potential impact of community-based TB/HIV screening and linkage to care over a 10-year period, we extended our previous model of TB and HIV transmission in a rural area of South Africa[^1^](#_ENREF_1) to include healthcare costs and years of life saved. We additionally updated the model parameters to reflect the most current data for TB and HIV incidence and treatment coverage.[^2^](#_ENREF_2)^,^[^3^](#_ENREF_3) We modeled drug-sensitive TB, MDR-TB, and XDR-TB, including acquired and amplified drug resistance, self-cure, strain fitness, exogenous re-infection, and mortality,[^4^](#_ENREF_4) and parameterized our model using data from clinical and epidemiological studies (Tables I and II).

In line with other models of TB infection,[^5^](#_ENREF_5) latent infections are stratified into rapid or slower progression. Individuals progress from latent infection to active infectious or non-infectious TB disease. Non-infectious individuals can become infectious over time,[^6^](#_ENREF_6)^,^[^7^](#_ENREF_7) and consistent with clinical studies, a small percentage of smear-negative patients are categorized as infectious.[^8^](#_ENREF_8) The effectiveness of TB treatment is determined by drug efficacy, adherence, and default.[^9^](#_ENREF_9) Individuals who receive effective treatment recover from active disease, while individuals who are ineffectively treated remain infectious and are at risk for acquired or amplified resistance.[^10^](#_ENREF_10) A small proportion of patients with active TB or ineffective treatment may also self-cure and return to a latently infected state.[^11^](#_ENREF_11) Patients who successfully complete treatment and recover may relapse to active disease.[^11^](#_ENREF_11) Both recovered and latently infected individuals can be exogenously re-infected with drug-sensitive or resistant TB, contingent upon partial immunity and the time-dependent risk of infection.[^12^](#_ENREF_12)

We modeled the early and late stages of HIV disease, mortality, and prevalence-dependent behavior change, as well as ART initiation and default. HIV-negative individuals can become infected with HIV at a rate proportional to the prevalence of HIV in the population.[^2^](#_ENREF_2)^,^[^13^](#_ENREF_13) Consistent with clinical recommendations in 2015, HIV-infected individuals with CD4+ cell count below 500 cells per milliliter are eligible to initiate ART,[^14^](#_ENREF_14) but only a proportion of actually begin ART.[^2^](#_ENREF_2)^,^[^13^](#_ENREF_13) HIV-infected individuals who are co-infected with active TB may also start ART, regardless of CD4+ cell count.[^14^](#_ENREF_14)

We take into account both the effect of HIV and ART on TB disease, as well as TB disease on HIV pathogenesis, such that disease progression and mortality rates depend on whether an individual is co-infected.[^15^](#_ENREF_15) Overall, HIV infection increases the likelihood of progression to primary active disease in those infected with TB, the rate at latent infection reactivates to active disease, and mortality from TB disease.[^16^](#_ENREF_16) Infection with HIV also reduces the efficacy of TB treatment, as well as partial immunity to superinfection or reinfection with another TB strain.[^16^](#_ENREF_16) Similarly, active TB disease decreases CD4+ cell counts, speeds up progression to AIDS, and increases mortality.[^15^](#_ENREF_15)^,^[^17^](#_ENREF_17) These exacerbations between TB and HIV are mitigated by ART.[^18-20^](#_ENREF_18)

**Intervention Strategies**

We compared the impact of community-based TB/HIV screening and linkage to care over a 10-year time horizon on the disease incidence, mortality, and healthcare costs arising from drug-sensitive TB, MDR-TB, XDR-TB and HIV. Specifically, we compared a total of eight mutually exclusive strategies (Table 1).

**Status quo TB and HIV detection and treatment**

We evaluated the effectiveness of community-based TB/HIV screening and linkage to care relative to status quo TB and HIV control currently implemented in rural South Africa. In the baseline scenario, individuals with TB and/or HIV symptoms self-present to a healthcare facility for symptom detection (*e.g.* cough lasting longer than two weeks, fever, night sweats, and/or weight loss), Xpert MTB/RIF (a newer, fully automated, cartridge-based technology to rapidly diagnose both TB disease and rifampicin resistance), sputum smear, and/or chest x-ray. We assume the status quo TB case detection rate to be 72.5%.[^2^](#_ENREF_2) The bacteriologic coverage (*i.e.* the percentage of suspected TB patients who receive microbiologic testing) for KwaZulu-Natal is estimated to be 80%.[^21^](#_ENREF_21) Per South African treatment guidelines,[^22^](#_ENREF_22) patients identified as having rifampicin-sensitive active TB disease through Xpert, as well as patients who do not receive microbiologic testing but are suspected of having active TB disease, are enrolled in first-line therapy (the administration of four oral antibiotics for two months and two antibiotics for at least an additional four months). Any TB patients remaining symptomatic and/or smear positive after two to three months of first-line therapy are suspected of TB drug resistance, then assessed by culture and drug susceptibility testing (DST).[^23^](#_ENREF_23) Although culture is the gold standard for diagnosis of active pulmonary TB disease, it can require over three months to receive the results of culture combined with DST.[^24^](#_ENREF_24)^,^[^25^](#_ENREF_25) Patients who have MDR or XDR-TB confirmed are then started on second-line therapy for at least 24 months. Patients who are initially identified through Xpert as having rifampicin-resistant active TB disease are also started on second-line therapy while awaiting confirmation by DST. Current second-line TB treatment guidelines recommend home-based treatment for MDR-TB management whenever possible, where patients are visited by a nurse for the administration of injectable drugs and supervision of oral medications over a 24-month period.[^26^](#_ENREF_26) XDR-TB patients are hospitalized for at least the intensive phase of their treatment.[^23^](#_ENREF_23)^,^[^26^](#_ENREF_26)

Following HIV diagnosis, individuals are referred to an HIV clinic to receive a CD4+ cell count to determine their eligibility for ART, but it is estimated that 15% to 45% of patients do not follow-up for this evaluation.[^27^](#_ENREF_27)^,^[^28^](#_ENREF_28) Of those who are determined to be ART-eligible (CD4+ cell count ≤ 500), only around half start treatment,[^28^](#_ENREF_28)^,^[^29^](#_ENREF_29) and about 25% of those individuals default within three years.[^30^](#_ENREF_30) We assume the current coverage of ART and IPT to be 52%.[^31-33^](#_ENREF_31) While the current South African guidelines recommend ART for individuals with a CD4+ cell count below 500 cells per milliliter, we additionally considered the scenario where all HIV-infected individuals were eligible for ART, regardless of CD4+ cell count, as has recently been demonstrated to be effective at reducing overall HIV incidence in a population.[^32^](#_ENREF_32)^,^[^33^](#_ENREF_33)

The South African Department of Health recommends 36 months of IPT for TST positive individuals and 12 months for TST negative or unknown individuals (from hereon forth abbreviated as 36/12 months)[^27^](#_ENREF_27)^,^[^34^](#_ENREF_34)^,^[^35^](#_ENREF_35) to treat latent infection and prevent progression to active TB.[^36^](#_ENREF_36) IPT is typically initiated simultaneously with ART.[^37^](#_ENREF_37)^,^[^38^](#_ENREF_38) We additionally considered the scenario where IPT was given to HIV-positive individuals for the entire duration of their lives, as has been recently recommended by the WHO.[^39^](#_ENREF_39) We assumed individuals treated with IPT and adherent to treatment were protected from infection with or latent reactivation of drug-susceptible TB for the duration of the IPT, dependent on treatment efficacy (22–100%) and patient adherence (21–87%), and that IPT does not prevent the reactivation or transmission of drug resistant TB.[^6^](#_ENREF_6)^,^[^35^](#_ENREF_35)^,^[^40^](#_ENREF_40) For the status quo scenario, we additionally assumed that only patients who initiated ART received IPT, as is currently implemented in South Africa,[^38^](#_ENREF_38) and that all patients on ART receive IPT. Upon IPT completion, individuals return to baseline relative risk of slower TB reactivation and infection.[^34^](#_ENREF_34)

**Community-based TB/HIV screening and linkage to care**

We model a community-based TB/HIV screening and linkage to care program that combines HIV testing and counseling with TB testing.[^1^](#_ENREF_1) TB/HIV screening is offered and administered to any individuals within the community who accept TB or HIV testing, with acceptance rates between 70% and 100%.[^41^](#_ENREF_41)^,^[^42^](#_ENREF_42) As the first step in this community-based intervention, a questionnaire is used to screen individuals for TB symptoms (sensitivity of 69–79%).[^43^](#_ENREF_43)^,^[^44^](#_ENREF_44) Sputum is then collected from symptomatic individuals for Xpert, culture, and DST (sensitivity of 68–100%).[^25^](#_ENREF_25) Individuals diagnosed by Xpert and/or culture are linked to the appropriate first- or second-line TB treatment, with 85% of individuals starting treatment.[^45^](#_ENREF_45)^,^[^46^](#_ENREF_46) Currently Xpert technology can only detect rifampicin resistance, and thus XDR-TB diagnoses can only be made after waiting two months for the DST results. We additionally considered the scenario where Xpert could detect resistance to second-line drugs and therefore be used to rapidly diagnose XDR-TB. Individuals are also given a rapid HIV antibody test (sensitivity of 98.2–100%).[^47^](#_ENREF_47) Those who test positive have their result confirmed by a second rapid HIV test, and then have a point-of-care CD4+ cell count performed to immediately determine their ART eligibility status. If eligible, patients are linked to their local ART clinic for ARV treatment and IPT initiation, with approximately 31% of individuals identified as eligible initiating ART and IPT.[^41^](#_ENREF_41)^,^[^42^](#_ENREF_42) We assume individuals with a CD4+ cell count less than 500 cells per milliliter to be eligible for ART, as has been recommended by South African ART guidelines.[^48^](#_ENREF_48) We additionally considered the scenario where all HIV-infected individuals were eligible for ART, irrespective of CD4+ cell count.[^32^](#_ENREF_32)^,^[^33^](#_ENREF_33)

Individuals not yet eligible for ART (with a CD4+ cell count greater than 500 cells per milliliter) are linked to their local ART clinic for IPT, as well as regular CD4+ cell count monitoring.[^41^](#_ENREF_41)^,^[^42^](#_ENREF_42) We considered two different approaches to IPT administration: a) 36/12 months of IPT, reflecting current South African guidelines for IPT administration; and b) lifetime IPT for all HIV patients, reflecting recent changes to WHO recommendations and findings that lifetime IPT may greatly reduce TB burden.[^39^](#_ENREF_39)^,^[^49^](#_ENREF_49)

The WHO states that there is not sufficient evidence to conclude that IPT administration increases MDR-TB incidence via the generation of isoniazid mono-resistance,[^39^](#_ENREF_39) so we assume that neither status quo IPT nor an increase in IPT coverage via the TB/HIV screening intervention will increase the incidence of MDR-TB. However, some predictions have been made that isoniazid mono-resistance may become highly prevalent after 50 or more years of IPT, thus increasing the burden of MDR and/or XDR-TB.[^6^](#_ENREF_6)^,^[^50^](#_ENREF_50) Although the time horizon for our analysis is only 10 years, in a sensitivity analysis we also consider the “worst case” scenario whereby the community-based TB/HIV screening immediately causes 100% of the modeled population to become resistant to isoniazid, thus decreasing the efficacy of first-line TB treatment from 77% to 65% and increasing the probability that first-line treatment failures acquire MDR-TB from 3.8% to 61%.[^51^](#_ENREF_51) While this worst case scenario is unrealistically pessimistic, and thus is not proposed as a likely possibility, it provides an upper bound on any potential increases in MDR- and XDR-TB incidence and thus on the costs that could arise from expanding IPT coverage.

Each modeled TB/HIV screening team consists of a professional nurse, two field health workers, and three counselors who screen between 1,800 and 4,800 individuals annually at community sites,[^1^](#_ENREF_1) costing between $61.83 and $23.18 per person screened, including the cost of a TB symptom questionnaire and rapid HIV test. We conservatively assume $61.83 per person screened as our base case cost, but vary the screening cost in the sensitivity analysis. In some settings, trained community health workers have been comparably effective at testing and linking individuals to HIV care as counselors, totaling as little as $6 per person screened.[^41^](#_ENREF_41) In the sensitivity analysis, we additionally vary the rates of linkage to care to reflect any differences that might arise in other settings. The South African Department of Health aims to screen all individuals for TB once per year,[^22^](#_ENREF_22) which we incorporate as the base case, but also consider screening frequencies of once every two years, once every year, and once every six months.

**Health outcomes and costs**

We considered the lifetime costs of TB and HIV detection and treatment among 90,000 adults in a rural South African setting. We performed the analysis over a 10-year time horizon from the perspective of the South African Department of Health, which is typically responsible for all medical costs in rural settings. Health burden was estimated with regard to the number of life years saved by an intervention strategy over the course of the lifetimes of the individuals in the population modeled. Costs and life years were discounted at an annual rate of 3%, following WHO guidelines.[^52^](#_ENREF_52) Costs were presented in 2015 US dollars (US$).

**Cost-effectiveness**

We calculated the incremental cost-effectiveness ratio (ICER) of the community-based TB/HIV screening and linkage to treatment strategies detailed above. The ICER of each strategy measures the additional cost per life year saved as the frequency of TB/HIV screening and/or the duration of IPT is increased. In accordance with WHO guidelines, we classified an intervention strategy as “very cost-effective” if its incremental cost-effectiveness ratio (ICER) was less than the South African per capita GDP in 2015 ($6,618) and as “cost-effective” if the ICER was less than three times the per capita GDP ($19,854).[^52^](#_ENREF_52)^,^[^53^](#_ENREF_53) Strategies with ICERs below the threshold for cost-effectiveness can be considered to be preferred or “economically efficient” strategies. Given that multiple strategies may be classified as cost-effective, the choice regarding which strategy should be implemented will ultimately depend on the Department of Health’s “willingness-to-pay” for each additional year of life saved. A strategy is considered “dominated,” (*i.e.* not optimal) if it costs more than an alternative strategy that is as or more effective.

**Uncertainty and Sensitivity Analysis**

To assess the impact of parameter uncertainty on our cost-effectiveness analysis and to estimate the likelihood that a strategy would be optimal at a given willingness-to-pay threshold, we performed a probabilistic sensitivity analysis.[^54^](#_ENREF_54) We assigned probability distributions to all parameters and costs by fitting the 95% confidence interval of beta or gamma distributions to plausible ranges from clinical and epidemiological data (ranges are shown in Tables I and II; uncertainty distributions are specified in Table III). Samples were drawn from these parameter distributions 1,000 times using Latin hypercube sampling and run through the model to project distributions of intervention cost and life years saved for each strategy. Net health benefits were calculated from the cost and life years saved distributions as the difference between the average health benefit of an intervention strategy (*i.e.* life years saved) and the average intervention and healthcare costs, divided by the threshold cost-effectiveness ratio.[^55^](#_ENREF_55) We calculated the net health benefit of each strategy across a range of willingness-to-pay thresholds. From this, we found the probability that a given scenario had the greatest net health benefit compared to its alternatives at a given level of willingness-to-pay. We used these probabilities to generate cost-effectiveness acceptability curves that quantified the uncertainty surrounding our cost-effectiveness ratio estimates. The acceptability curves demonstrate the likelihood that a given intervention strategy saved the largest number of life years for a given outlay (*i.e.* was optimal at a given willingness-to-pay threshold).

Considering South Africa’s goal of screening all individuals once per year, we also performed a one-way sensitivity analysis to determine the impact of parameters on the cost-effectiveness ratio of annual TB/HIV screening. Specifically, we calculated the ICER of annual TB/HIV screening with 36/12 months of IPT and lifetime IPT at the minimum and maximum value of each parameter (Tables I and II) to determine whether the strategy remained cost-effective at these extreme values.

**Model Equations**

Our model consists of differential equations, characterizing joint TB and HIV transmission in a rural South African community. The modeled disease states and parameters are listed in Tables II and IV. The model diagrams of TB and HIV infection, diagnosis, and treatment are depicted in Figures I-III. For clarity, here we describe TB and HIV transmission separately, where *X* corresponds to the TB disease state; *Y* corresponds to the HIV disease state; *i* = infectious or noninfectious TB; and *j* = DS (*j*=1), MDR (*j*=2), or XDR (*j*=3) TB; *^T^* = TB; *^H^* = HIV. However, the model incorporates both TB and HIV disease states simultaneously.

**Drug Susceptible TB**

**MDR-TB**

**XDR-TB**

**HIV**

**Force of Infection**

**References**

1. Gilbert JA, Long EF, Brooks RP, et al. Integrating Community-Based Interventions to Reverse the Convergent TB/HIV Epidemics in Rural South Africa. *PLoS One* 2015; **10**(5): e0126267.

2. WHO. WHO TB Burden Estimates. 2014.

3. Statistics South Africa. Mid-year population estimates 2014. Pretoria, South Africa, 2014.

4. Basu S, Andrews JR, Poolman EM, et al. Prevention of nosocomial transmission of extensively drug-resistant tuberculosis in rural South African district hospitals: an epidemiological modelling study. *Lancet* 2007; **370**(9597): 1500-7.

5. Dye C, Williams BG. Criteria for the control of drug-resistant tuberculosis. *Proceedings of the National Academy of Sciences of the United States of America* 2000; **97**(14): 8180-5.

6. Cohen T, Lipsitch M, Walensky RP, Murray M. Beneficial and perverse effects of isoniazid preventive therapy for latent tuberculosis infection in HIV-tuberculosis coinfected populations. *Proceedings of the National Academy of Sciences of the United States of America* 2006; **103**(18): 7042-7.

7. Cohen T, Murray M. Modeling epidemics of multidrug-resistant M. tuberculosis of heterogeneous fitness. *Nature medicine* 2004; **10**(10): 1117-21.

8. Hernandez-Garduno E, Cook V, Kunimoto D, Elwood RK, Black WA, FitzGerald JM. Transmission of tuberculosis from smear negative patients: a molecular epidemiology study. *Thorax* 2004; **59**(4): 286-90.

9. WHO. Global Tuberculosis Report 2013. Geneva, Switzerland, 2013.

10. Mukherjee JS, Rich ML, Socci AR, et al. Programmes and principles in treatment of multidrug-resistant tuberculosis. *Lancet* 2004; **363**(9407): 474-81.

11. Dye C, Garnett GP, Sleeman K, Williams BG. Prospects for worldwide tuberculosis control under the WHO DOTS strategy. Directly observed short-course therapy. *Lancet* 1998; **352**(9144): 1886-91.

12. Cohen T, Colijn C, Finklea B, Murray M. Exogenous re-infection and the dynamics of tuberculosis epidemics: local effects in a network model of transmission. *Journal of the Royal Society, Interface / the Royal Society* 2007; **4**(14): 523-31.

13. Actuarial Society of South Africa. ASSA2008 Model. 2011.

14. WHO. Antiretroviral therapy for HIV infection in adults and adolescents. Recommendations for a public health approach. 2010 revision. Geneva, Switzerland: World Health Organization, 2010.

15. Martin DJ, Sim JG, Sole GJ, et al. CD4+ lymphocyte count in African patients co-infected with HIV and tuberculosis. *Journal of acquired immune deficiency syndromes and human retrovirology : official publication of the International Retrovirology Association* 1995; **8**(4): 386-91.

16. WHO. WHO Three I's Meeting. Intensified Case Finding (ICF), Isoniazid Preventive Therapy (IPT) and TB Infection Control (IC) for people living with HIV. Geneva, Switzerland: World Health Organization, 2008.

17. Modjarrad K, Vermund SH. Effect of treating co-infections on HIV-1 viral load: a systematic review. *The Lancet infectious diseases* 2010; **10**(7): 455-63.

18. Badri M, Wilson D, Wood R. Effect of highly active antiretroviral therapy on incidence of tuberculosis in South Africa: a cohort study. *Lancet* 2002; **359**(9323): 2059-64.

19. Golub JE, Saraceni V, Cavalcante SC, et al. The impact of antiretroviral therapy and isoniazid preventive therapy on tuberculosis incidence in HIV-infected patients in Rio de Janeiro, Brazil. *AIDS* 2007; **21**(11): 1441-8.

20. Lawn SD, Kranzer K, Wood R. Antiretroviral therapy for control of the HIV-associated tuberculosis epidemic in resource-limited settings. *Clin Chest Med* 2009; **30**(4): 685-99, viii.

21. Health Systems Trust. Bacteriologic Coverage Rate (%). 2012. <http://indicators.hst.org.za/healthstats/164/data> (accessed 7 April 2015.

22. Republic of South Africa. National Tuberculosis Management Guidelines. South Africa, 2014.

23. Basu S, Friedland GH, Medlock J, et al. Averting epidemics of extensively drug-resistant tuberculosis. *Proceedings of the National Academy of Sciences of the United States of America* 2009; **106**(18): 7672-7.

24. WHO. Laboratory services in tuberculosis control. Part III: culture. Geneva, Switzerland: WHO, 1998.

25. Dowdy DW, Chaisson RE, Maartens G, Corbett EL, Dorman SE. Impact of enhanced tuberculosis diagnosis in South Africa: a mathematical model of expanded culture and drug susceptibility testing. *Proceedings of the National Academy of Sciences of the United States of America* 2008; **105**(32): 11293-8.

26. Republic of South Africa. Multi-Drug Resistant Tuberculosis. A Policy Framework on Decentralised and Deinstitutionalised Management for South Africa, 2011.

27. Republic of South Africa. The South African Antiretroviral Treatment Guidelines, 2013.

28. Rosen S, Fox MP. Retention in HIV care between testing and treatment in sub-Saharan Africa: a systematic review. *PLoS medicine* 2011; **8**(7): e1001056.

29. Bassett IV, Regan S, Chetty S, et al. Who starts antiretroviral therapy in Durban, South Africa?... not everyone who should. *AIDS* 2010; **24 Suppl 1**: S37-44.

30. Fox MP, Rosen S. Patient retention in antiretroviral therapy programs up to three years on treatment in sub-Saharan Africa, 2007-2009: systematic review. *Tropical medicine & international health : TM & IH* 2010; **15 Suppl 1**: 1-15.

31. Johnson LF. Access to Antiretroviral Treatment in South Africa, 2004-2011. *The Southern African Journal of HIV Medicine* 2012; **13**(1): 22-7.

32. Group TAS. A Trial of Early Antiretrovirals and Isoniazid Preventive Therapy in Africa. *N Engl J Med* 2015.

33. Group ISS. Initiation of Antiretroviral Therapy in Early Asymptomatic HIV Infection. *N Engl J Med* 2015.

34. Houben RM, Sumner T, Grant AD, White RG. Ability of preventive therapy to cure latent Mycobacterium tuberculosis infection in HIV-infected individuals in high-burden settings. *Proceedings of the National Academy of Sciences of the United States of America* 2014; **111**(14): 5325-30.

35. WHO. Guidelines for intensified tuberculosis case-finding and isoniazid preventive therapy for people living with HIV in resource-constrained settings. Geneva, Switzerland: WHO, 2011.

36. WHO. WHO Policy on Collaborative TB/HIV Activities: Guidelines for National Programmes and Other Stakeholders. Geneva, Switzerland, 2012.

37. Chehab JC, Vilakazi-Nhlapo K, Vranken P, Peters A, Klausner JD. Survey of isoniazid preventive therapy in South Africa, 2011. *The international journal of tuberculosis and lung disease : the official journal of the International Union against Tuberculosis and Lung Disease* 2012; **16**(7): 903-7.

38. Churchyard GJ, Mametja LD, Mvusi L, et al. Tuberculosis control in South Africa: Successes, challenges and recommendations. *S Afr Med J* 2014; **104**(3 (Suppl 1)): 244-8.

39. WHO. Recommendation on 36 months of isoniazid preventive therapy to adults and adolescents living with HIV in resource-constrained and high TB- and HIV-prevalence settings: 2015 update: WHO, 2015.

40. Basu S, Maru D, Poolman E, Galvani A. Primary and secondary tuberculosis preventive treatment in HIV clinics: simulating alternative strategies. *The international journal of tuberculosis and lung disease : the official journal of the International Union against Tuberculosis and Lung Disease* 2009; **13**(5): 652-8.

41. Smith JA, Sharma M, Levin C, et al. Cost-effectiveness of community-based strategies to strengthen the continuum of HIV care in rural South Africa: a health economic modelling analysis. *Lancet HIV* 2015; **2**(4): e159-e68.

42. Bassett IV, Govindasamy D, Erlwanger AS, et al. Mobile HIV screening in Cape Town, South Africa: clinical impact, cost and cost-effectiveness. *PLoS One* 2014; **9**(1): e85197.

43. Ayles H, Schaap A, Nota A, et al. Prevalence of tuberculosis, HIV and respiratory symptoms in two Zambian communities: implications for tuberculosis control in the era of HIV. *PLoS One* 2009; **4**(5): e5602.

44. Getahun H, Kittikraisak W, Heilig CM, et al. Development of a standardized screening rule for tuberculosis in people living with HIV in resource-constrained settings: individual participant data meta-analysis of observational studies. *PLoS medicine* 2011; **8**(1): e1000391.

45. Dowdy DW, O'Brien MA, Bishai D. Cost-effectiveness of novel diagnostic tools for the diagnosis of tuberculosis. *The international journal of tuberculosis and lung disease : the official journal of the International Union against Tuberculosis and Lung Disease* 2008; **12**(9): 1021-9.

46. Loveday M, Wallengren K, Voce A, et al. Comparing early treatment outcomes of MDR-TB in decentralised and centralised settings in KwaZulu-Natal, South Africa. *The international journal of tuberculosis and lung disease : the official journal of the International Union against Tuberculosis and Lung Disease* 2012; **16**(2): 209-15.

47. Branson DM. Point-of-Care Rapid Tests for HIV Antibodies. *J Lab Med* 2003; **27**: 288-95.

48. Republic of South Africa Department of Health. National consolidated guidelines for the prevention of mother-to-child transmission of HIV (PMTCT) and the management of HIV in children, adolescents and adults. Pretoria, South Africa: Department of Health, Republic of South Africa, 2014.

49. Lawn SD, Wood R, De Cock KM, Kranzer K, Lewis JJ, Churchyard GJ. Antiretrovirals and isoniazid preventive therapy in the prevention of HIV-associated tuberculosis in settings with limited health-care resources. *The Lancet infectious diseases* 2010; **10**(7): 489-98.

50. Mills HL, Cohen T, Colijn C. Community-wide isoniazid preventive therapy drives drug-resistant tuberculosis: a model-based analysis. *Science translational medicine* 2013; **5**(180): 180ra49.

51. Jacobson KR, Theron D, Victor TC, Streicher EM, Warren RM, Murray MB. Treatment outcomes of isoniazid-resistant tuberculosis patients, Western Cape Province, South Africa. *Clinical infectious diseases : an official publication of the Infectious Diseases Society of America* 2011; **53**(4): 369-72.

52. WHO. Macroeconomics and health: investing in health for economic development. Geneva, 2001.

53. World Bank. GDP per capita (current US$). 2013. <http://data.worldbank.org/indicator/NY.GDP.PCAP.CD> (accessed 11 May 2015 2015).

54. Briggs AH, Weinstein MC, Fenwick EA, et al. Model parameter estimation and uncertainty analysis: a report of the ISPOR-SMDM Modeling Good Research Practices Task Force Working Group-6. *Med Decis Making* 2012; **32**(5): 722-32.

55. Stinnett AA, Mullahy J. Net health benefits: a new framework for the analysis of uncertainty in cost-effectiveness analysis. *Med Decis Making* 1998; **18**(2 Suppl): S68-80.

56. WHO. Global Tuberculosis Report 2014. Geneva, Switzerland: WHO, 2014.

57. Boehme CC, Nicol MP, Nabeta P, et al. Feasibility, diagnostic accuracy, and effectiveness of decentralised use of the Xpert MTB/RIF test for diagnosis of tuberculosis and multidrug resistance: a multicentre implementation study. *Lancet* 2011; **377**(9776): 1495-505.

58. Gandhi NR, Moll A, Sturm AW, et al. Extensively drug-resistant tuberculosis as a cause of death in patients co-infected with tuberculosis and HIV in a rural area of South Africa. *Lancet* 2006; **368**(9547): 1575-80.

59. South Africa Department of Health. The KZN "outbreak". Pretoria: DoH, 2006.

60. Orenstein EW, Basu S, Shah NS, et al. Treatment outcomes among patients with multidrug-resistant tuberculosis: systematic review and meta-analysis. *The Lancet infectious diseases* 2009; **9**(3): 153-61.

61. Jacobson KR, Tierney DB, Jeon CY, Mitnick CD, Murray MB. Treatment outcomes among patients with extensively drug-resistant tuberculosis: systematic review and meta-analysis. *Clinical infectious diseases : an official publication of the Infectious Diseases Society of America* 2010; **51**(1): 6-14.

62. Health Systems Trust. Defaulter (interruption) rate (new Sm+ cases) (%). 2011. <http://indicators.hst.org.za/healthstats/167/data> (accessed 21 April 2015 2015).

63. Mlambo CK, Warren RM, Poswa X, Victor TC, Duse AG, Marais E. Genotypic diversity of extensively drug-resistant tuberculosis (XDR-TB) in South Africa. *The international journal of tuberculosis and lung disease : the official journal of the International Union against Tuberculosis and Lung Disease* 2008; **12**(1): 99-104.

64. Wallengren K, Scano F, Nunn P, et al. Drug-Resistant tuberculosis, KwaZulu-Natal, South Africa, 2001-2007. *Emerging infectious diseases* 2011; **17**(10): 1913-6.

65. Pooran A, Pieterson E, Davids M, Theron G, Dheda K. What is the cost of diagnosis and management of drug resistant tuberculosis in South Africa? *PLoS One* 2013; **8**(1): e54587.

66. Menzies NA, Cohen T, Lin HH, Murray M, Salomon JA. Population Health Impact and Cost-Effectiveness of Tuberculosis Diagnosis with Xpert MTB/RIF: A Dynamic Simulation and Economic Evaluation. *PLoS medicine* 2012; **9**(11): e1001347.

67. Cleary S, Okorafor O, Chitha W, Boulle A, Jikwana S. South African Health Reveiw: Chapter 5 - Financing Antiretroviral Treatment and Primary Health Care Services. Durban: Health Systems Trust, 2005.

68. Bassett IV, Giddy J, Nkera J, et al. Routine voluntary HIV testing in Durban, South Africa: the experience from an outpatient department. *J Acquir Immune Defic Syndr* 2007; **46**(2): 181-6.

69. Granich R, Kahn JG, Bennett R, et al. Expanding ART for treatment and prevention of HIV in South Africa: estimated cost and cost-effectiveness 2011-2050. *PLoS One* 2012; **7**(2): e30216.

70. Cleary S, Boulle A, McIntyre D, Coetzee D. Cost-Effectiveness of Antiretroviral Treatment for HIV-Positive Adults in a South African Township. Cape Town, South Africa: University of Cape Town, 2004.

71. Akolo C, Adetifa I, Shepperd S, Volmink J. Treatment of latent tuberculosis infection in HIV infected persons. *Cochrane Database Syst Rev* 2010; (1): CD000171.

72. Gupta S, Abimbola T, Date A, et al. Cost-effectiveness of the Three I's for HIV/TB and ART to prevent TB among people living with HIV. *The international journal of tuberculosis and lung disease : the official journal of the International Union against Tuberculosis and Lung Disease* 2014; **18**(10): 1159-65.

73. Govindasamy D, Kranzer K, van Schaik N, et al. Linkage to HIV, TB and non-communicable disease care from a mobile testing unit in Cape Town, South Africa. *PLoS One* 2013; **8**(11): e80017.

74. van Rooyen H, Barnabas RV, Baeten JM, et al. High HIV testing uptake and linkage to care in a novel program of home-based HIV counseling and testing with facilitated referral in KwaZulu-Natal, South Africa. *J Acquir Immune Defic Syndr* 2013; **64**(1): e1-8.

75. Lawn SD, Harries AD, Williams BG, et al. Antiretroviral therapy and the control of HIV-associated tuberculosis. Will ART do it? *The international journal of tuberculosis and lung disease : the official journal of the International Union against Tuberculosis and Lung Disease* 2011; **15**(5): 571-81.

76. Havlir DV, Getahun H, Sanne I, Nunn P. Opportunities and challenges for HIV care in overlapping HIV and TB epidemics. *JAMA : the journal of the American Medical Association* 2008; **300**(4): 423-30.

77. WHO. Treatment of Tuberculosis Guidelines. Fourth edition. Geneva, Switzerlnd: WHO, 2010.

78. Health Systems Trust. Defaulter (interruption) rate (new Sm+ cases) (%). 2013. <http://indicators.hst.org.za/healthstats/167/data> (accessed 30 April 2013.

79. Holtz TH, Lancaster J, Laserson KF, Wells CD, Thorpe L, Weyer K. Risk factors associated with default from multidrug-resistant tuberculosis treatment, South Africa, 1999-2001. *The international journal of tuberculosis and lung disease : the official journal of the International Union against Tuberculosis and Lung Disease* 2006; **10**(6): 649-55.

80. Shean KP, Willcox PA, Siwendu SN, et al. Treatment outcome and follow-up of multidrug-resistant tuberculosis patients, West Coast/Winelands, South Africa, 1992-2002. *The international journal of tuberculosis and lung disease : the official journal of the International Union against Tuberculosis and Lung Disease* 2008; **12**(10): 1182-9.

81. Gandhi NR, Moll AP, Lalloo U, et al. Successful integration of tuberculosis and HIV treatment in rural South Africa: the Sizonq'oba study. *J Acquir Immune Defic Syndr* 2009; **50**(1): 37-43.

82. Long EF, Brandeau ML, Owens DK. The cost-effectiveness and population outcomes of expanded HIV screening and antiretroviral treatment in the United States. *Annals of internal medicine* 2010; **153**(12): 778-89.

83. Boehme C, Nicol M, Nabeta P, et al. Feasibility, diagnostic accuracy, and effectiveness of decentralised use of the Xpert MTB/RIF test for diagnosis of tuberculosis and multidrug resistance: a multicentre implementation study. *Lancet* 2011; **377**(9776): 1495-505.

84. Health Systems Trust. Bacteriological coverage rate (%. 2013. <http://indicators.hst.org.za/healthstats/164/data> (accessed 4 June 2013.

85. Cobelens FG, Egwaga SM, van Ginkel T, Muwinge H, Matee MI, Borgdorff MW. Tuberculin skin testing in patients with HIV infection: limited benefit of reduced cutoff values. *Clinical infectious diseases : an official publication of the Infectious Diseases Society of America* 2006; **43**(5): 634-9.

86. Republic of South Africa. National Consolidated Guidelines for the Prevention of Mother-to-Child Transmission of HIV (PMTCT) and the Management of HIV in Children, Adolescents and Adults. South Africa: Department of Health, 2014.

**Table I.** **Additional epidemiological model parameters, ranges, and references.** Rates are in units of year^-1^ unless otherwise noted. *The extent to which ART mitigates the effect of HIV on the natural history of TB is quantified by the parameter *k.*

| Parameter | Symbol | TB Disease Status | | HIV– | HIV+ (No ART) | HIV+ (ART) | Reference |
| --- | --- | --- | --- | --- | --- | --- | --- |
| Annual population growth rate | *g* |  | | 0.0584 (0.04–0.06) | NA | NA | [^1^](#_ENREF_1)^,^[^2^](#_ENREF_2)^,^[^13^](#_ENREF_13) |
| Annual mortality rate | *μ* | *Baseline*  *(no TB)* | | 0.008  (0.004–0.015) | 0.008 (0.004–0.015, CD4+  >500),  0.17 (0.1–0.5, CD4+  ≤500) | 0.008 (0.004–0.015, CD4+  >500),  0.056 (0.015–0.30, CD4+  ≤500) | [^1^](#_ENREF_1)^,^[^2^](#_ENREF_2)^,^[^4^](#_ENREF_4)^,^[^13^](#_ENREF_13)^,^[^15^](#_ENREF_15)^,^[^17^](#_ENREF_17)^,^[^25^](#_ENREF_25)^,^[^75^](#_ENREF_75)^,^[^76^](#_ENREF_76) |
|  | *μ_TB_* | *Active TB* | | 0.30 (0.2–0.4) | 0.992 (0.75–1) | 0.508 (0.2–0.76) | [^1^](#_ENREF_1)^,^[^2^](#_ENREF_2)^,^[^4^](#_ENREF_4)^,^[^13^](#_ENREF_13) |
| TB transmissibility coefficient | *β_TB_* |  | | 8.65 x 10^-5^  (8 x 10^-5^–1 x 10^-4^) | 8.65 x 10^-5^  (8 x 10^-5^–1 x 10^-4^) | 8.65 x 10^-5^  (8 x 10^-5^–1 x 10^-4^) | [^1^](#_ENREF_1)^,^[^2^](#_ENREF_2)^,^[^13^](#_ENREF_13) |
| Relative fitness penalty drug resistance TB (proportion) | *h_2_* | *MDR* | | 0.12  (0–0.7) | 0.12  (0–0.7) | 0.12  (0–0.7) | [^4^](#_ENREF_4)^,^[^7^](#_ENREF_7) |
|  | *h_3_* | *XDR* | | 0.16  (0–0.7) | 0.16  (0–-0.7) | 0.16  (0–0.7) | [^4^](#_ENREF_4)^,^[^7^](#_ENREF_7) |
| Proportion who develop primary progressive TB | *p* |  | | 0.14  (0.08–0.25) | 0.67  (0.36–0.8) | * | [^4^](#_ENREF_4)^,^[^23^](#_ENREF_23) |
| Endogenous annual TB reactivation rate | *ν* | *Long latency* | | 0.00011 (0.0001–0.0003) | 0.17 (0.04–0.2) | * | [^4^](#_ENREF_4)^,^[^23^](#_ENREF_23) |
|  | *τ* | *Short latency* | | 0.88 (0.76–0.99) | 12 (10.4–13.5) | * | [^4^](#_ENREF_4)^,^[^23^](#_ENREF_23) |
| Degree susceptibility despite prior TB infection (proportion) | *x_TB_* |  | | 0.35 (0.1–0.6) | 0.75 (0.5–1) | * | [^4^](#_ENREF_4) |
| Proportion of TB infections that are infectious | *f* |  | | 0.65 (0.5–0.65) | 0.3 (0.19–0.4) | * | [^4^](#_ENREF_4) |
| Rate of conversion from non-infectious to infectious TB | *w* |  | | 0.015 (0.007–0.02) | 0.015 (0.007–0.02) | 0.015 (0.007–0.02) | [^4^](#_ENREF_4)^,^[^11^](#_ENREF_11) |
| Rate of natural self cure | *ζ* |  | | 0.2 (0.15–0.25) | 0.2 (0.15–0.25) | 0.2 (0.15–0.25) | [^4^](#_ENREF_4) |
| Baseline TB detection and treatment | *δ* |  | | 1.2917  (0.9163–2.3026) | 1.2917  (0.9163–2.3026) | 1.2917  (0.9163–2.3026) | [^1^](#_ENREF_1)^,^[^2^](#_ENREF_2)^,^[^13^](#_ENREF_13) |
| Proportion cured by 1^st^ line TB drugs | *q_11_* | *DS* | | 0.73 (0.65–0.88) | 0.58 (0.52–0.74) | * | [^4^](#_ENREF_4)^,^[^7^](#_ENREF_7) |
|  | *q_12_* | *MDR* | | 0.47 (0.18–0.58) | 0.30 (0.16–0.36) | * | [^4^](#_ENREF_4)^,^[^7^](#_ENREF_7)^,^[^58^](#_ENREF_58)^,^[^59^](#_ENREF_59) |
|  | *q_13_* | *XDR* | | 0 | 0 | 0 | [^4^](#_ENREF_4)^,^[^7^](#_ENREF_7)^,^[^58^](#_ENREF_58)^,^[^59^](#_ENREF_59) |
| Proportion cured by 2^nd^ line TB drugs | *q_22_* | *MDR* | | 0.67 (0.43–0.80) | 0.45 (0.43–0.51) | * | [^4^](#_ENREF_4)^,^[^7^](#_ENREF_7)^,^[^58-60^](#_ENREF_58) |
|  | *q_33_* | *XDR* | | 0.54 (0.328–0.545) | 0.36 (0.328–0.400) | * | [^4^](#_ENREF_4)^,^[^58^](#_ENREF_58)^,^[^61^](#_ENREF_61) |
| Duration TB treatment (months) | $\frac{1}{\rho_{1}}$ | *DS* | | 6 (6–8) | 6 (6–8) | 6 (6–8) | [^77^](#_ENREF_77) |
|  | $\frac{1}{\rho_{2}}$ | *MDR* | | 24 (18–24) | 24 (18–24) | 24 (18–24) | [^26^](#_ENREF_26) |
|  | $\frac{1}{\rho_{3}}$ | *XDR* | | 26 (24.6–27.8) | 26 (24.6–27.8) | 26 (24.6–27.8) | [^23^](#_ENREF_23) |
| Proportion defaulting from TB treatment | *d_TB1_* | *DS* | | 0.07 (0.048–0.088) | 0.07 (0.048–0.088) | 0.07 (0.048–0.088) | [^78^](#_ENREF_78) |
|  | *d_TB2_* | *MDR*  *outpatient* | | 0.07 (0.048–0.088) | 0.07 (0.048–0.088) | 0.07 (0.048–0.088) | [^64^](#_ENREF_64)^,^[^79^](#_ENREF_79)^,^[^80^](#_ENREF_80) |
|  | *d_TB3_* | *MDR/XDR*  *inpatient* | | 0.28 (0.19–0.37) | 0.28 (0.19–0.37) | 0.28 (0.19–0.37) | [^26^](#_ENREF_26)^,^[^63^](#_ENREF_63)^,^[^64^](#_ENREF_64) |
| Proportion of treated TB patients who acquire or amplify resistance | *ω_1_* | *DS to MDR* | | 0.038 (0.025–0.1) | 0.038 (0.025–0.1) | 0.038 (0.025–0.1) | [^4^](#_ENREF_4) |
|  | *ω_2_* | *MDR to XDR* | | 0.030 (0.025–0.1) | 0.030 (0.025–0.1) | 0.030 (0.025–0.1) | [^4^](#_ENREF_4) |
| Time to identification of TB treatment failure (months) | $\frac{1}{\kappa}$ |  | | 2 (1–4) | 2 (1–4) | 2 (1–4) | [^23^](#_ENREF_23) |
| Time from culture collection to MDR treatment initiation (days) | $\frac{1}{\sigma_{DS}}$ | *Decentralized* | | 72  (56–99) | 72  (56–99) | 72  (56–99) | [^46^](#_ENREF_46) |
| Proportion of cases defaulting before TB treatment initiation | *l_TB_* | *DS/MDR outpatient (DOTS)* | | 0.15 (0–0.24) | 0.15 (0–0.24) | 0.15 (0–0.24) | [^45^](#_ENREF_45) |
|  |  | *MDR/XDR inpatient* | | 0.5 (0.29–0.73) | 0.5 (0.29–0.73) | 0.5 (0.29–0.73) | [^26^](#_ENREF_26) |
|  |  |  |  |  |  |  |  |
| HIV transmissibility coefficient | *β_HIV_* |  | | NA | 1.67x10^-6^ (1 x 10^-6^–2 x 10^-6^) | 1.67x10^-6^  (1 x 10^-6^–2 x 10^-6^) | [^1^](#_ENREF_1)^,^[^2^](#_ENREF_2)^,^[^13^](#_ENREF_13) |
| α (HIV transmission shape parameter) | *α* |  | | NA | 4.31 (1–6) | 4.31 (1–6) | [^1^](#_ENREF_1)^,^[^2^](#_ENREF_2)^,^[^13^](#_ENREF_13) |
| Relative infectivity of HIV on ART (proportion) | *x_HIV_* |  | | NA | NA | 0.12 (0–0.12) | [^81^](#_ENREF_81) |
| Relative infectivity of ART eligible | *v* |  | | NA | 2.92 (1.00–3.33) | NA | [^1^](#_ENREF_1)^,^[^2^](#_ENREF_2)^,^[^13^](#_ENREF_13)^,^[^82^](#_ENREF_82) |
| ART coverage (CD4+ < 500) | *η* |  | | NA | 52% (20–80%) | NA | [^31^](#_ENREF_31) |
| Proportion of cases detected defaulting before HIV treatment initiation | *l_HIV_* | *Screening Program* | NA | | 0.69  (0.16–0.8) | NA | [^41^](#_ENREF_41)^,^[^42^](#_ENREF_42)^,^[^73^](#_ENREF_73)^,^[^74^](#_ENREF_74) |
| Proportion retained in HIV treatment over 3 years | *d_HIV_* |  | | NA | NA | 0.75 (0.64–0.87) | [^30^](#_ENREF_30) |
| Rate of relapse from chemotherapeutic cure | *υ* |  | | 0.001 (0–0.01) | 0.001 (0–0.01) | 0.001 (0–0.01) | [^4^](#_ENREF_4) |
| Duration of HIV infection, CD4+ cell count >500 (years) | $\frac{1}{\gamma}$ |  | | NA | 4 (3–5); 2 (1–3, active TB infx) | NA | [^15^](#_ENREF_15)^,^[^17^](#_ENREF_17)^,^[^75^](#_ENREF_75)^,^[^76^](#_ENREF_76) |
| Sensitivity of TB symptom screening | *m* |  | | 69% (51.9–83.7%) | 79% (58.3–90.9%) | 79% (58.3–90.9%) | [^43^](#_ENREF_43)^,^[^44^](#_ENREF_44) |
| Sensitivity of TB sputum culture/DST | *c_i_* | *Infectious* | | 100% | 100% | 100% | [^25^](#_ENREF_25) |
|  | *c_n_* | *Noninfectious* | | 68% | 68% | 68% | [^25^](#_ENREF_25) |
| Sensitivity of Xpert MTB/RIF for TB diagnosis | *b_XPTi_* | *Infectious* | | 98.3% (97–99%) | 98.3% (97–99%) | 98.3% (97–99%) | [^83^](#_ENREF_83) |
|  | *b_XPTn_* | *Noninfectious* | | 76.9% (72.4–80.8%) | 76.9% (72.4–80.8%) | 76.9% (72.4–80.8%) | [^83^](#_ENREF_83) |
| Sensitivity of Xpert MTB/RIF for RIF resistance detection given MTB detected | *y* |  | | 94.4% (90.8–98.6%) | 94.4% (90.8–98.6%) | 94.4% (90.8–98.6%) | [^83^](#_ENREF_83) |
| Sensitivity of rapid HIV antibody test | *r* |  | | NA | 100% (98.2–100%) | NA | [^47^](#_ENREF_47) |
| Bacteriologic coverage rate | *t* |  | | 80% (80–100%) | 80% (80–100%) | 80% (80–100%) | [^84^](#_ENREF_84) |
| Percentage of HIV+ individuals who are TST+ | *s* |  | | NA | 70% (64.3–74.5%) | 70% (64.3–74.5%) | [^85^](#_ENREF_85) |
| IPT efficacy for drug-sensitive TB | *e* |  | | NA | 100% (22–100%) | 100% (22–100%) | [^6^](#_ENREF_6)^,^[^40^](#_ENREF_40) |
| IPT adherence | *Y* |  | | NA | 87% (21–87%) | 87% (21–87%) | [^40^](#_ENREF_40)^,^[^71^](#_ENREF_71) |
| Duration of IPT (months) | *ψ* |  | | NA | 6 (TST negative or unknown, standard duration); lifelong (lifelong duration) | 6 (TST negative or unknown, standard duration); lifelong (lifelong duration) | [^86^](#_ENREF_86) |
|  | *ψ_TST_* |  | | NA | 36 (TST positive, standard duration); lifelong (lifelong duration) | 36 (TST positive, standard duration); lifelong (lifelong duration) | [^86^](#_ENREF_86) |
| Default from IPT | *d_IPT_* |  | | NA | 0.091 (4–0.5) | 0.091 (4–0.5) | [^30^](#_ENREF_30) |
| Effectiveness of ART in reversing effect of HIV on TB natural history | *k* |  | | NA | NA | 0.7 (0.47–0.87) | [^18-20^](#_ENREF_18) |

**Table II. Uncertainty distributions for model and cost parameters.** All rates and probabilities are annual. The 95% confidence interval of gamma or beta distributions were fit to the parameter ranges in Table 1 and Table I. The shape *a* and scale *b* gamma distribution parameters are specified as Gamma(*a*,*b*), while the first shape *a* and second shape *b* beta distribution parameters are specified as Beta(*a*,*b*). *The extent to which ART mitigates the effect of HIV on the natural history of TB is quantified by the parameter *k.*

| Parameter | Symbol | TB Disease Status | | HIV– | HIV+ (No ART) | HIV+ (ART) | Reference |
| --- | --- | --- | --- | --- | --- | --- | --- |
| Annual population growth rate | *g* |  | | Gamma(94, 0.0005) | NA | NA | [^1^](#_ENREF_1)^,^[^2^](#_ENREF_2)^,^[^13^](#_ENREF_13) |
| Annual mortality rate | *μ* | *Baseline*  *(no TB)* | | Gamma(9, 9 x 10^-4^) | Gamma(9, 9 x 10^-4^) (CD4+  >500),  Gamma(6, 0.04) (CD4+  ≤500) | Gamma(9, 9 x 10^-4^) (CD4+  >500),  * (CD4+  ≤500) | [^1^](#_ENREF_1)^,^[^2^](#_ENREF_2)^,^[^4^](#_ENREF_4)^,^[^13^](#_ENREF_13)^,^[^15^](#_ENREF_15)^,^[^17^](#_ENREF_17)^,^[^25^](#_ENREF_25)^,^[^75^](#_ENREF_75)^,^[^76^](#_ENREF_76) |
|  | *μ_TB_* | *Active TB* | | Gamma(32, 0.008) | Gamma(186, 0.005) | Gamma(9, 0.05) | [^1^](#_ENREF_1)^,^[^2^](#_ENREF_2)^,^[^4^](#_ENREF_4)^,^[^13^](#_ENREF_13) |
| TB transmissibility coefficient | *β_TB_* |  | | Gamma(309,3 x 10^-7^) | Gamma(309, 3 x 10^-7^) | Gamma(309, 3 x 10^-7^) | [^1^](#_ENREF_1)^,^[^2^](#_ENREF_2)^,^[^13^](#_ENREF_13) |
| Relative fitness penalty drug resistance TB (proportion) | *h_2_* | *MDR* | | Beta(2, 4) | Beta(2, 4) | Beta(2, 4) | [^4^](#_ENREF_4)^,^[^7^](#_ENREF_7) |
|  | *h_3_* | *XDR* | | Beta(2, 4) | Beta(2, 4) | Beta(2, 4) | [^4^](#_ENREF_4)^,^[^7^](#_ENREF_7) |
| Proportion who develop primary progressive TB | *p* |  | | Beta(11, 57) | Beta(10, 7) | * | [^4^](#_ENREF_4)^,^[^23^](#_ENREF_23) |
| Endogenous annual TB reactivation rate | *ν* | *Long latency* | | Gamma(13, 1 x 10^-5^) | Gamma(6, 0.02) | * | [^4^](#_ENREF_4)^,^[^23^](#_ENREF_23) |
|  | *τ* | *Short latency* | | Gamma(220, 0.004) | Gamma(226, 0.05) | * | [^4^](#_ENREF_4)^,^[^23^](#_ENREF_23) |
| Degree susceptibility despite prior TB infection (proportion) | *x_TB_* |  | | Beta(4, 8) | Beta(6, 2) | * | [^4^](#_ENREF_4) |
| Proportion of TB infections that are infectious | *f* |  | | Beta(95, 70) | Beta(20, 50) | * | [^4^](#_ENREF_4) |
| Rate of conversion from non-infectious to infectious TB | *w* |  | | Gamma(14, 0.0009) | Gamma(14, 0.0009) | Gamma(14, 0.0009) | [^4^](#_ENREF_4)^,^[^11^](#_ENREF_11) |
| Rate of natural self cure | *ζ* |  | | Gamma(59, 0.003) | Gamma(59, 0.003) | Gamma(59, 0.003) | [^4^](#_ENREF_4) |
| Baseline TB detection and treatment | *δ* |  | | Gamma(22, 0.07) | Gamma(22, 0.07) | Gamma(22, 0.07) | [^1^](#_ENREF_1)^,^[^2^](#_ENREF_2)^,^[^13^](#_ENREF_13) |
| Proportion cured by 1^st^ line TB drugs | *q_11_* | *DS* | | Beta(38, 11) | Beta(46, 26) | * | [^4^](#_ENREF_4)^,^[^7^](#_ENREF_7) |
|  | *q_12_* | *MDR* | | Beta(8, 13) | Beta(18, 53) | * | [^4^](#_ENREF_4)^,^[^7^](#_ENREF_7)^,^[^58^](#_ENREF_58)^,^[^59^](#_ENREF_59) |
| Proportion cured by 2^nd^ line TB drugs | *q_22_* | *MDR* | | Beta(15, 9) | Beta(280, 316) | * | [^4^](#_ENREF_4)^,^[^7^](#_ENREF_7)^,^[^58-60^](#_ENREF_58) |
|  | *q_33_* | *XDR* | | Beta(34, 44) | Beta(34, 44) | * | [^4^](#_ENREF_4)^,^[^58^](#_ENREF_58)^,^[^61^](#_ENREF_61) |
| Duration TB treatment | $\rho_{1}$ | *DS* | | Gamma(186, 0.009) | Gamma(186, 0.009) | Gamma(186, 0.009) | [^77^](#_ENREF_77) |
|  | $\rho_{2}$ | *MDR* | | Gamma(186, 0.003) | Gamma(186, 0.003) | Gamma(186, 0.003) | [^26^](#_ENREF_26) |
|  | $\rho_{3}$ | *XDR* | | Gamma(1028, 0.0004) | Gamma(1028, 0.0004) | Gamma(1028, 0.0004) | [^23^](#_ENREF_23) |
| Proportion defaulting from TB treatment | *d_TB1_* | *DS* | | Beta(40, 554) | Beta(40, 554) | Beta(40, 554) | [^78^](#_ENREF_78) |
|  | *d_TB2_* | *MDR*  *outpatient* | | Beta(40, 554) | Beta(40, 554) | Beta(40, 554) | [^64^](#_ENREF_64)^,^[^79^](#_ENREF_79)^,^[^80^](#_ENREF_80) |
|  | *d_TB3_* | *MDR/XDR*  *inpatient* | | Beta(26, 67) | Beta(26, 67) | Beta(26, 67) | [^26^](#_ENREF_26)^,^[^63^](#_ENREF_63)^,^[^64^](#_ENREF_64) |
| Proportion of treated TB patients who acquire or amplify resistance | *ω_1_* | *DS to MDR* | | Beta(8, 134) | Beta(8, 134) | Beta(8, 134) | [^4^](#_ENREF_4) |
|  | *ω_2_* | *MDR to XDR* | | Beta(8, 134) | Beta(8, 134) | Beta(8, 134) | [^4^](#_ENREF_4) |
| Time to identification of TB treatment failure | $\kappa$ |  | | Gamma(8, 0.8) | Gamma(8, 0.8) | Gamma(8, 0.8) | [^23^](#_ENREF_23) |
| Time from culture collection to MDR treatment initiation | $\sigma_{DS}$ | *Decentralized* | | Gamma(48, 0.1) | Gamma(48, 0.1) | Gamma(48, 0.1) | [^46^](#_ENREF_46) |
| Proportion of cases defaulting before TB treatment initiation | *l_TB_* | *DS/MDR outpatient (DOTS)* | | Beta(2, 2) | Beta(2, 2) | Beta(2, 2) | [^45^](#_ENREF_45) |
|  |  | *MDR/XDR inpatient* | | Beta(9, 9) | Beta(9, 9) | Beta(9, 9) | [^26^](#_ENREF_26) |
|  |  |  |  |  |  |  |  |
| HIV transmissibility coefficient | *β_HIV_* |  | | NA | Gamma(32, 4x10^-8^) | Gamma(32, 4x10^-8^) | [^1^](#_ENREF_1)^,^[^2^](#_ENREF_2)^,^[^13^](#_ENREF_13) |
| α (HIV transmission shape parameter) | *α* |  | | NA | Gamma(5, 0.6) | Gamma(5, 0.6) | [^1^](#_ENREF_1)^,^[^2^](#_ENREF_2)^,^[^13^](#_ENREF_13) |
| Relative infectivity of HIV on ART (proportion) | *x_HIV_* |  | | NA | NA | Beta(2, 37) | [^81^](#_ENREF_81) |
| Relative infectivity of ART eligible | *v* |  | | NA | Gamma(11, 0.2) | NA | [^1^](#_ENREF_1)^,^[^2^](#_ENREF_2)^,^[^13^](#_ENREF_13)^,^[^82^](#_ENREF_82) |
| ART coverage (CD4+ < 500) | *η* |  | | NA | Gamma(156, 0.005) | NA | [^31^](#_ENREF_31) |
| Proportion of cases detected defaulting before HIV treatment initiation | *l_HIV_* | *Screening Program* | NA | | Beta(8, 15) | NA | [^41^](#_ENREF_41)^,^[^42^](#_ENREF_42)^,^[^73^](#_ENREF_73)^,^[^74^](#_ENREF_74) |
| Proportion retained in HIV treatment over 3 years | *d_HIV_* |  | | NA | NA | Gamma(12, 0.008) | [^30^](#_ENREF_30) |
| Rate of relapse from chemotherapeutic cure | *υ* |  | | Gamma(1, 0.003) | Gamma(1, 0.003) | Gamma(1, 0.003) | [^4^](#_ENREF_4) |
| Duration of HIV infection, CD4+ cell count >500 | $\gamma$ |  | | NA | Gamma(59, 0.004) (no TB), Gamma(13, 0.05) (TB disease) | NA | [^15^](#_ENREF_15)^,^[^17^](#_ENREF_17)^,^[^75^](#_ENREF_75)^,^[^76^](#_ENREF_76) |
| Sensitivity of TB symptom screening | *m* |  | | Beta(21, 10) | Beta(18, 6) | Beta(18, 6) | [^43^](#_ENREF_43)^,^[^44^](#_ENREF_44) |
| Sensitivity of Xpert MTB/RIF for TB diagnosis | *b_XPTi_* | *Infectious* | | Beta(681, 13) | Beta(681, 13) | Beta(681, 13) | [^83^](#_ENREF_83) |
|  | *b_XPTn_* | *Noninfectious* | | Beta(297, 90) | Beta(297, 90) | Beta(297, 90) | [^83^](#_ENREF_83) |
| Sensitivity of Xpert MTB/RIF for RIF resistance detection given MTB detected | *y* |  | | Beta(99, 5) | Beta(99, 5) | Beta(99, 5) | [^83^](#_ENREF_83) |
| Sensitivity of rapid HIV antibody test | *r* |  | | NA | Beta(165, 1) | NA | [^47^](#_ENREF_47) |
| Bacteriologic coverage rate | *t* |  | | Beta(29, 5) | Beta(29, 5) | Beta(29, 5) | [^84^](#_ENREF_84) |
| Percentage of HIV+ individuals who are TST+ | *s* |  | | NA | Beta(216, 95) | Beta(216, 95) | [^85^](#_ENREF_85) |
| IPT efficacy for drug-sensitive TB | *e* |  | | NA | Beta(3, 2) | Beta(3, 2) | [^6^](#_ENREF_6)^,^[^40^](#_ENREF_40) |
| IPT adherence | *Y* |  | | NA | Beta(0.6, 0.03) | Beta(0.6, 0.03) | [^40^](#_ENREF_40)^,^[^71^](#_ENREF_71) |
| Default from IPT | *d_IPT_* |  | | NA | Gamma(4, 0.05) | Gamma(4, 0.05) | [^30^](#_ENREF_30) |
| Effectiveness of ART in reversing effect of HIV on TB natural history | *k* |  | | NA | NA | Beta(11, 5) | [^18-20^](#_ENREF_18) |
| Acceptance of TB/HIV Screening |  |  | | Beta(1, 0.02) | Beta(1, 0.02) | Beta(1, 0.02) | [^41^](#_ENREF_41)^,^[^42^](#_ENREF_42)^,^[^73^](#_ENREF_73)^,^[^74^](#_ENREF_74) |
| TB/HIV Screening Costs (monthly) | | *TB positive and HIV positive* | | NA | Gamma(17, 10) | NA | [^41^](#_ENREF_41)^,^[^42^](#_ENREF_42)^,^[^65^](#_ENREF_65) |
|  |  | *TB positive* | | Gamma(17, 8) | NA | Gamma(17, 8) | [^41^](#_ENREF_41)^,^[^42^](#_ENREF_42)^,^[^65^](#_ENREF_65) |
|  |  | *HIV positive* | | NA | Gamma(10, 5) | NA | [^41^](#_ENREF_41)^,^[^42^](#_ENREF_42)^,^[^65^](#_ENREF_65) |
|  |  | *TB negative and HIV negative* | | Gamma(6, 4) | NA | NA | [^41^](#_ENREF_41)^,^[^42^](#_ENREF_42)^,^[^65^](#_ENREF_65) |
| IPT costs (monthly) | |  | | NA | Gamma(4, 9 x 10^-7^) | Gamma(4, 9 x 10^-7^) | [^72^](#_ENREF_72) |
| Status quo HIV diagnosis (per patient) | |  | | NA | Gamma(295, 0.09) | NA | [^42^](#_ENREF_42)^,^[^67^](#_ENREF_67) |
| HIV treatment and healthcare costs (monthly) | | *Not in healthcare* | | NA | Gamma(54, 1) | NA | [^67-70^](#_ENREF_67) |
|  |  | *In healthcare, not on ART* | | NA | Gamma(150, 0.8) | NA | [^67-70^](#_ENREF_67) |
|  |  | *In healthcare, on ART* | | NA | NA | Gamma(31, 4) | [^67-70^](#_ENREF_67) |
| Status quo TB diagnosis (per patient) | | *Baseline screening* | | Gamma(23, 2) | Gamma(23, 2) | Gamma(23, 2) | [^65^](#_ENREF_65)^,^[^66^](#_ENREF_66) |
|  |  | *MDR/XDR-TB suspect* | | Gamma(22, 7) | Gamma(22, 7) | Gamma(22, 7) | [^65^](#_ENREF_65)^,^[^66^](#_ENREF_66) |
| TB treatment and healthcare costs (monthly) | | *First-line TB treatment and healthcare* | | Gamma(5, 0.006) | Gamma(5, 0.006) | Gamma(5, 0.006) | [^65^](#_ENREF_65)^,^[^66^](#_ENREF_66) |
|  |  | *MDR-TB, second-line TB treatment and healthcare* | | Gamma(44, 5) | Gamma(44, 5) | Gamma(44, 5) | [^65^](#_ENREF_65)^,^[^66^](#_ENREF_66) |
|  |  | *XDR-TB, second-line TB treatment and healthcare* | | Gamma(29, 26) | Gamma(29, 26) | Gamma(29, 26) | [^65^](#_ENREF_65)^,^[^66^](#_ENREF_66) |
| Specificity Symptom Questionnaire | |  | | Beta(0.9, 0.02) | Beta(0.7, 0.04) | Beta(0.7, 0.04) | [^43^](#_ENREF_43)^,^[^44^](#_ENREF_44) |
| Specificity Xpert for TB detection | |  | | Beta(2, 0.009) | Beta(2, 0.009) | Beta(2, 0.009) | [^57^](#_ENREF_57) |
| Specificity Xpert for rifampicin resistance detection | |  | | Beta(1, 0.009) | Beta(1, 0.009) | Beta(1, 0.009) | [^57^](#_ENREF_57) |
| Specificity rapid HIV test | |  | | NA | Beta(223, 2) | NA | [^47^](#_ENREF_47) |

**Table III. Epidemiological Impacts.** Epidemiological impact of community-based TB/HIV screening and linkage to care at frequencies of once every 2 years (Screen 2 yr), 1 year (Screen 1 yr), and 6 months (Screen 6 mo) relative to status quo with 36/12 months of IPT (IPT 36/12) or lifelong IPT (life IPT) on total TB incidence, HIV incidence (%), MDR-TB incidence, and XDR-TB incidence over 10 years.

|  | **2015** | **2016** | **2017** | **2018** | **2019** | **2020** | **2021** | **2022** | **2023** | **2024** | **2025** |
| --- | --- | --- | --- | --- | --- | --- | --- | --- | --- | --- | --- |
| **TB incidence per 100,000** |  |  |  |  |  |  |  |  |  |  |  |
| **Status Quo, IPT 36/12** | 868 | 713 | 598 | 533 | 485 | 447 | 417 | 392 | 371 | 353 | 338 |
| **Screen 2 yr, IPT 36/12** | 868 | 689 | 550 | 479 | 430 | 395 | 368 | 347 | 329 | 313 | 299 |
| **Screen 1 yr, IPT 36/12** | 868 | 667 | 509 | 435 | 389 | 357 | 333 | 314 | 298 | 284 | 272 |
| **Screen 6 mo, IPT 36/12** | 868 | 629 | 445 | 373 | 331 | 305 | 285 | 270 | 257 | 246 | 235 |
| **Status Quo, IPT life** | 868 | 706 | 569 | 486 | 427 | 384 | 351 | 326 | 306 | 290 | 276 |
| **Screen 2 yr, IPT life** | 868 | 679 | 510 | 414 | 351 | 309 | 280 | 259 | 243 | 231 | 2211 |
| **Screen 1 yr, IPT life** | 868 | 655 | 461 | 359 | 299 | 261 | 236 | 219 | 206 | 197 | 189 |
| **Screen 6 mo, IPT life** | 868 | 613 | 387 | 286 | 233 | 204 | 186 | 175 | 167 | 160 | 155 |
| **HIV incidence (%)** |  |  |  |  |  |  |  |  |  |  |  |
| **Status Quo, IPT 36/12** | 1.0 | 1.0 | 1.0 | 1.0 | 1.0 | 0.9 | 0.9 | 0.9 | 0.9 | 0.9 | 0.8 |
| **Screen 2 yr, IPT 36/12** | 1.0 | 1.0 | 1.0 | 0.9 | 0.9 | 0.9 | 0.8 | 0.8 | 0.8 | 0.8 | 0.8 |
| **Screen 1 yr, IPT 36/12** | 1.0 | 1.0 | 0.9 | 0.9 | 0.8 | 0.8 | 0.8 | 0.8 | 0.8 | 0.7 | 0.7 |
| **Screen 6 mo, IPT 36/12** | 1.0 | 1.0 | 0.9 | 0.8 | 0.8 | 0.7 | 0.7 | 0.7 | 0.7 | 0.7 | 0.7 |
| **Status Quo, IPT life** | 1.0 | 1.0 | 1.0 | 1.0 | 1.0 | 0.9 | 0.9 | 0.9 | 0.9 | 0.9 | 0.8 |
| **Screen 2 yr, IPT life** | 1.0 | 1.0 | 1.0 | 0.9 | 0.9 | 0.8 | 0.8 | 0.8 | 0.8 | 0.8 | 0.7 |
| **Screen 1 yr, IPT life** | 1.0 | 1.0 | 0.9 | 0.8 | 0.8 | 0.8 | 0.7 | 0.7 | 0.7 | 0.7 | 0.7 |
| **Screen 6 mo, IPT life** | 1.0 | 1.0 | 0.8 | 0.8 | 0.7 | 0.7 | 0.7 | 0.6 | 0.6 | 0.6 | 0.6 |
| **MDR-TB incidence per 100,000** |  |  |  |  |  |  |  |  |  |  |  |
| **Status Quo, IPT 36/12** | 55 | 46 | 36 | 31 | 27 | 25 | 23 | 21 | 20 | 19 | 18 |
| **Screen 2 yr, IPT 36/12** | 55 | 45 | 35 | 29 | 26 | 23 | 21 | 20 | 19 | 18 | 17 |
| **Screen 1 yr, IPT 36/12** | 55 | 44 | 33 | 28 | 24 | 22 | 20 | 19 | 17 | 17 | 16 |
| **Screen 6 mo, IPT 36/12** | 55 | 43 | 31 | 26 | 22 | 20 | 18 | 17 | 16 | 15 | 14 |
| **Status Quo, IPT life** | 55 | 45 | 36 | 30 | 27 | 24 | 22 | 21 | 20 | 19 | 18 |
| **Screen 2 yr, IPT life** | 55 | 45 | 34 | 28 | 24 | 22 | 20 | 19 | 18 | 17 | 16 |
| **Screen 1 yr, IPT life** | 55 | 44 | 33 | 27 | 23 | 21 | 19 | 17 | 16 | 16 | 15 |
| **Screen 6 mo, IPT life** | 55 | 42 | 30 | 24 | 21 | 19 | 17 | 16 | 15 | 14 | 13 |
| **XDR-TB incidence per 100,000** |  |  |  |  |  |  |  |  |  |  |  |
| **Status Quo, IPT 36/12** | 12 | 12 | 10 | 9 | 8 | 7 | 7 | 7 | 6 | 6 | 6 |
| **Screen 2 yr, IPT 36/12** | 12 | 11 | 10 | 8 | 8 | 7 | 7 | 6 | 6 | 6 | 6 |
| **Screen 1 yr, IPT 36/12** | 12 | 11 | 9 | 8 | 7 | 7 | 6 | 6 | 6 | 5 | 5 |
| **Screen 6 mo, IPT 36/12** | 12 | 11 | 9 | 7 | 7 | 6 | 6 | 5 | 5 | 5 | 5 |
| **Status Quo, IPT life** | 12 | 12 | 10 | 8 | 8 | 7 | 7 | 6 | 6 | 6 | 6 |
| **Screen 2 yr, IPT life** | 12 | 11 | 9 | 8 | 7 | 6 | 6 | 6 | 5 | 5 | 5 |
| **Screen 1 yr, IPT life** | 12 | 11 | 9 | 7 | 7 | 6 | 6 | 5 | 5 | 5 | 5 |
| **Screen 6 mo, IPT life** | 12 | 11 | 8 | 7 | 6 | 5 | 5 | 5 | 5 | 4 | 4 |

**Table IV. Modeled Disease States.**

| **Disease State** | **Description** |
| --- | --- |
| **TB** | |
| *S* | Susceptible |
| *P_S_* | Susceptible receiving IPT |
| *L* | Latent infection, slow |
| *E* | Latent infection, fast |
| *P_L_* | Latent infection receiving IPT |
| *A* | Active infection |
| *T* | Active infection on effective TB treatment |
| *F* | Active infection in ineffective TB treatment |
| *D* | Active infection detected and awaiting culture/DST results |
| *R* | Recovered |
| *P_R_* | Recovered receiving IPT |
| HIV | |
| *S* | Susceptible |
| *I* | Infected, CD4+ cell count > 500 cells/ml |
| *E* | Infected, CD4+ cell count ≤ 500 cells/ml |
| *I_Care_* | Infected, not on ART, in HIV care at a CD4+ cell count > 500 cells/ml |
| *I_ART_* | Infected and on ART at CD4+ cell count > 500 cells/ml |
| *E_ART_* | Infected and on ART at CD4+ cell count ≤ 500 cells/ml |

**Figure I. Model diagrams.** Model diagrams of (A) TB transmission dynamics and (B) HIV transmission dynamics. Although depicted separately, modeled individuals had a status for each disease. Drug resistance (*i.e.*, drug susceptible [DS], multidrug-resistant [MDR], and extensively drug-resistant [XDR] TB) was also included in the model. Model parameters and equations are detailed in S1 Text.

**Figure II. TB Diagnosis and Treatment Pathway Diagrams**. Flow diagram depicting TB diagnosis and treatment within the model for (A) community-based TB/HIV screening and (B) standard of care.

**A**

**B**

**Figure III. HIV Diagnosis and Treatment Pathway Diagrams**. Flow diagram depicting HIV diagnosis and treatment within the model for (A) community-based TB/HIV screening and (B) standard of care.

**A**

**B**

**Figure IV. Epidemiological impact when ART eligibility expanded to all HIV-infected individuals.** Impact of annual community-based TB/HIV screening and linkage to care (Annual Screen) relative to status quo (Status Quo) with 36/12 months of IPT (IPT 36/12) or lifelong IPT (IPT life) on (A) total TB incidence, (B) HIV incidence (%), (C) MDR-TB incidence, and (D) XDR-TB incidence over 10 years when ART eligibility expanded to all individuals with HIV.

**A**

**B**

**C**

**D**
